# HER2 quantitative continuous scoring for accurate patient selection in HER2 negative trastuzumab deruxtecan treated breast cancer

**DOI:** 10.1038/s41598-024-61957-9

**Published:** 2024-05-27

**Authors:** Ansh Kapil, Andreas Spitzmüller, Nicolas Brieu, Susanne Haneder, Anatoliy Shumilov, Armin Meier, Fabiola Cecchi, Alice Barkell, Nathalie Harder, Katrin Mittermaier, Ana Hidalgo-Sastre, Regina Alleze, Markus Schick, Günter Schmidt, Hadassah Sade, Zenta Tsuchihashi, Fumitaka Suto, Mark Gustavson, J. Carl Barrett, Danielle Carroll

**Affiliations:** 1 Computational Pathology, Oncology R&D, AstraZeneca, Bernhard-Wicki-Straße 5, 80636 München, Bayern Germany; 2 Translational Medicine, Oncology R&D, AstraZeneca, Gaithersburg, MD USA; 3 Translational Medicine, Oncology R&D, AstraZeneca, Cambridge, UK; 4 Translational Science, Daiichi Sankyo, Inc., Basking Ridge, NJ USA

**Keywords:** Tumour biomarkers, Computational models, Tumour heterogeneity

## Abstract

Many targeted cancer therapies rely on biomarkers assessed by scoring of immunohistochemically (IHC)-stained tissue, which is subjective, semiquantitative, and does not account for expression heterogeneity. We describe an image analysis-based method for quantitative continuous scoring (QCS) of digital whole-slide images acquired from baseline human epidermal growth factor receptor 2 (HER2) IHC-stained breast cancer tissue. Candidate signatures for patient stratification using QCS of HER2 expression on subcellular compartments were identified, addressing the spatial distribution of tumor cells and tumor-infiltrating lymphocytes. Using data from trastuzumab deruxtecan-treated patients with HER2-positive and HER2-negative breast cancer from a phase 1 study (NCT02564900; DS8201-A-J101; N = 151), QCS-based patient stratification showed longer progression-free survival (14.8 vs 8.6 months) with higher prevalence of patient selection (76.4 vs 56.9%) and a better cross-validated log-rank *p* value (0.026 vs 0.26) than manual scoring based on the American Society of Clinical Oncology / College of American Pathologists guidelines. QCS-based features enriched the HER2-negative subgroup by correctly predicting 20 of 26 responders.

## Introduction

Invasive breast cancer (BC) is the most common cancer in women worldwide. Human epidermal growth factor receptor 2 (HER2) protein is overexpressed in 20 to 25% of breast carcinomas^1^. According to the established 2018 American Society of Clinical Oncology (ASCO)/College of American Pathologists (CAP) guidelines^2^, HER2 positivity is defined by either (a) >10% of tumor cells with intense, complete, and circumferential membranous HER2 immunohistochemistry (IHC) staining patterns (IHC 3+) or (b) weak to moderate membrane staining observed in >10% of tumor cells (IHC 2+) and coupled with a confirmation of *HER2* gene amplification. Prior to anti-HER2 targeted therapies, patients with HER2-positive BC had more aggressive disease, higher recurrence rates, and increased mortality^3^.

**Figure 1:**
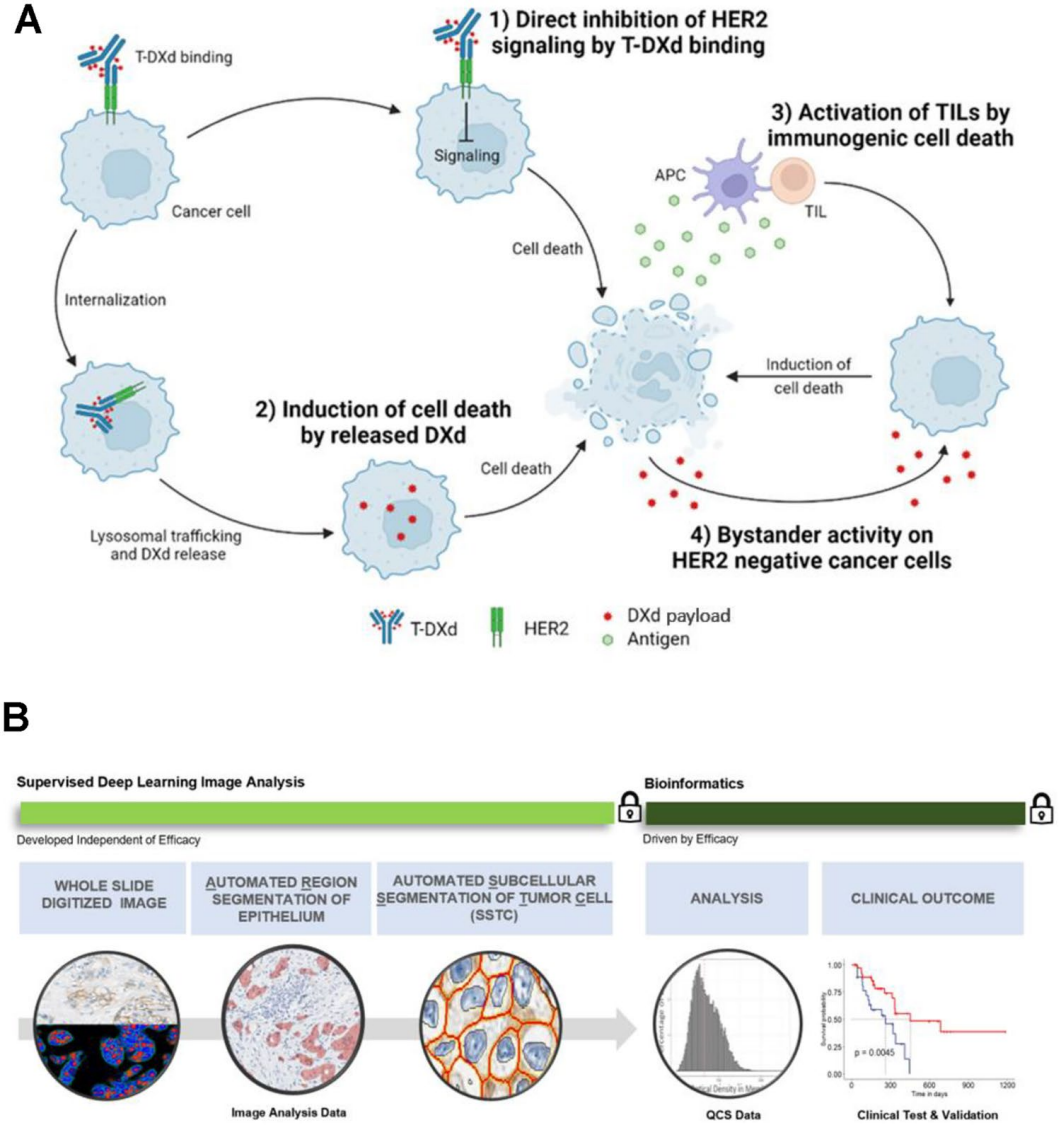
(**A**) Illustration of the hypothetical mechanisms of action of trastuzumab deruxtecan (T-DXd). (1) Binding of the antibody-drug conjugate (ADC) causes cell damage via a dysfunctional human epidermal growth factor receptor 2 (HER2) pathway. (2) Internalization of the ADC, subsequent linker cleavage, and diffusion of the released topoisomerase I inhibitor payload into the nucleus causes cell death by DNA damage. (3) Tumor antigens released by apoptotic cancer cells are recognized by the host immune system, which may induce systemic immunogenic cell death. (4) Topoisomerase I inhibitor released from apoptotic cancer cells may kill bystander cells. *TIL* tumor-infiltrating lymphocyte. (**B**) Workflow of the proposed quantitative continuous scoring (QCS). Padlock indicates the model was locked.

Trastuzumab deruxtecan (T-DXd) is a newer, HER2-directed humanized antibody-drug conjugate (ADC). T-DXd comprises an immunoglobulin G1 monoclonal antibody with the same amino acid sequence as trastuzumab, a cleavable linker, and a topoisomerase I inhibitor (deruxtecan) payload, conjugated via thioether bonds to the reduced cysteine residues found in the antibody. As illustrated in Fig. 1A, the dominant mechanism of action (MOA) is associated with the internalization of the ADC and its subsequent intracellular payload release. As evaluated by pharmacodynamic studies^4^, the internalization rate is driven, in part, by the expression level of HER2 protein in the membrane. A second ADC-specific MOA, called the bystander antitumor effect, results from the high cell membrane permeability of the payload, which enables not only the ADC-specific killing of target-positive cells but also target-negative cells in their proximity^5^. Given its unique properties and MOA, T-DXd has shown antitumor activity, not only in patients with HER2-positive (IHC 3+, IHC 2+/in situ hybridization [ISH]+)^6–8^ but also in the newly characterized HER2-low category (IHC 1+ or 2+/ISH−) of HER2-expressing BC^9^ tumors.

Patients with HER2-positive BC are considered suitable for treatment with targeted anti-HER2 therapies such as trastuzumab^1^. The existing ASCO/CAP HER2 scoring guidelines^2^ and the approved IHC assays were designed to aid the selection of patients who are most likely to benefit from drugs that block the cell signaling pathways downstream of HER2, specifically trastuzumab^2^. However, the current guidelines do not accommodate the bystander effect of ADCs (Fig. 1A). Therefore, as further data emerge on ADC efficacy in lower levels of HER2 expression, more biomarker information may be useful to identify patients who may benefit from ADCs, relying not only on a targeted action but also on potential bystander effects. Given the additional MOA of T-DXd and its proven efficacy in tumors with low levels of HER2 expression^9^, there is a challenge in appropriately identifying patients who may benefit. Standard or adapted assays were sufficient for identifying patients who were HER2-positive or HER2-low, respectively. With the inherent challenges of IHC and low levels of expression, alternative biomarker scoring strategies may be needed to optimize the selection of patients most likely to benefit. We explore new methods of interpretation of the existing strategies using advanced approaches such as deep-learning–based image and survival data analysis to better quantify the biology of HER2 expression and model response to therapy as a function of HER2 expression.

We demonstrate the use of deep-learning–based image analysis (IA) of digitized tissue sections as a more sensitive, highly granular, and quantitative approach to quantify HER2 protein levels of expression throughout its entire spectrum, allowing for an improved patient stratification in T-DXd–treated BC samples. We showed that, besides patients with highly HER2-positive scores, this approach is able to identify more patients who may benefit from T-DXd therapy, including those with low (IHC 1+, IHC 2+/ISH−) or IHC >0 to <1+ HER2 scores, a heterogeneous tumor with respect to single-cell HER2 expression, or a high density of tumor-infiltrating lymphocytes (TILs). In contrast to other IA approaches aimed at equivalence with pathologist scoring that may lead to a US Food and Drug Administration 510(k) approval^3^, our method builds on the accurate segmentation of individual tumor cells and their respective membrane, cytoplasmic, and nuclear compartments, and on the detection of TILs. The precise delineation of these objects of interest enables us to estimate the amount of expressed HER2 protein for each tumor cell in the tissue sections on a subcellular level. The amount of HER2 protein expression is characterized in terms of optical density (OD) in the membrane and the cytoplasm of each tumor cell. On a patient level, this information, the mean OD per subcellular compartment, is represented as a histogram that describes the distribution of OD values per patient. Combined with the spatial distribution statistics of the cell populations, we obtain a rich set of features that characterize the patient’s quantitative continuous scoring (QCS) status (Fig. 1B). Furthermore, to model the unique MOA of T-DXd—the potential bystander activity of the drug—we developed the spatial proximity score (SPS) to capture spatial heterogeneity and its effect on patient survival.

To test the clinical relevance of these QCS features, we retrospectively analyzed data from a phase 1 clinical trial of T-DXd (NCT02564900; DS8201-A-J101 [J101])^10^ comprising data from 186 patients with previously treated HER2-positive and HER2-negative BC. In the retrospective analysis, we showed that QCS-based biomarkers are superior to manual pathological HER2 assessment to select patients who are likely to benefit from T-DXd therapy.

## Results

### J101 clinical trial details

J101 was an open-label, two-part, multicenter study to evaluate the safety and tolerability of T-DXd in patients with advanced solid malignant tumors^8^. The study consisted of two parts: part 1 (dose escalation) and part 2 (dose expansion). Within this study, a total of 186 patients with previously treated HER2-positive and HER2-negative (HER2-low [IHC 1+, IHC 2+/ISH–] and IHC 0) BC were analyzed. In the current analysis, all BC samples from dose escalation as well as the dose-expansion phase (parts 2a, 2c, and 2e), for which digitized images were available, were considered.

**Figure 2:**
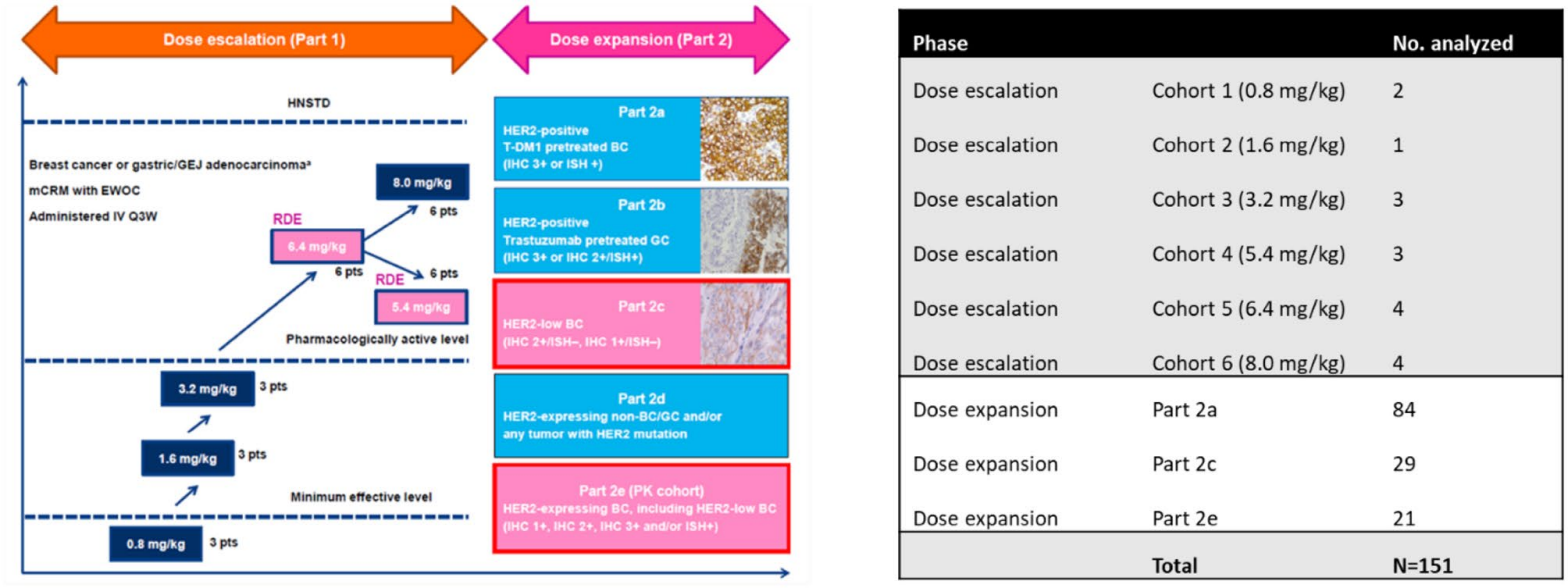
The study design of the NCT02564900 (DS8201-A-J101) clinical trial. *BC* breast cancer, *EWOC* escalation with overdose control, *GC* gastric cancer, *GEJ* gastroesophageal junction adenocarcinoma, *HER2* human epidermal growth factor receptor 2, *IHC* immunohistochemistry, *ISH* in situ hybridization, *IV* intravenous, *mCRM* modified continual reassessment method, *PK* pharmacokinetic, *pts* patients, *Q3W* every 3 weeks, *RDE* recommended dose for expansion, *T*-*DM1* trastuzumab emtansine.

HER2 IHC (HercepTest; Dako)-stained, whole-slide images (WSIs) were available for a subset of 151 patients (Fig. 2). Associated ASCO/CAP HER2 pathologist scores, demographic data, and clinical data, including overall and progression-free survival (PFS), were available. All samples were rescored at a central laboratory according to ASCO/CAP HER2 guidelines, and some discordance was observed with local laboratory results. The central laboratory scores were used for this analysis. Even though the patient population in this trial was skewed toward HER2-high expression, there was a subset of patients with centrally confirmed HER2-negative status (n = 65: including HER2-low [IHC 1+, n = 39 or IHC 2+/ISH−, n = 17] and HER2 IHC 0, n = 9) enrolled and treated in this clinical trial. In the whole analyzed patient cohort, the centrally confirmed objective response rate (ORR) defined as complete response or partial response according to Response Evaluation Criteria in Solid Tumors version 1.1 was 50%, with 76 responders and 75 non-responders. The median PFS was 13.7 months. In the centrally confirmed HER2-negative subset (n = 65), a median PFS of 11 months was observed, and 26 of 65 patients (40.0%) responded to T-DXd.

### QCS-image analysis results from J101

To confirm the performance of the QCS-IA algorithms (details in Materials and methods), analytical testing was performed on unseen WSIs that were not used for training or model selection. The ground truth for analytical testing was obtained from annotations of carefully selected regions of interest (ROIs) from multiple pathologists (N=3). QCS-IA results were compared against the concordance of multiple pathologists for the following metrics during analytical testing: (1) epithelial region segmentation (dice score), (2) cell center detection (F1 scores), (3) membrane segmentation (average symmetric surface distance), and (4) 3,3-diaminobenzidine (DAB) OD values from segmented membranes (Pearson correlation). The ROIs were chosen to capture the diversity of tissue morphology and staining heterogeneity across the different cohorts. From the development cohorts (see “Materials and methods”), a total of 116 ROIs were selected. The results are summarized in Table 1A. All the IA metrics apart from the membrane average symmetric surface distance were found to be in a similar range (±5%) as those of the concordance of multiple pathologists’ annotations. However, this average subpixel error on membrane segmentation did not contribute to large errors on subsequent membrane OD measurements, as seen by the strong Pearson correlation of ODs between QCS-IA and pathologists’ annotations. After confirmation of the QCS-IA model performance, the model was locked and blindly applied to the J101 clinical trial images without any modifications or retraining on J101 images.

**Table 1:** Image segmentation test results for QCS algorithm - Epithelial detection, cell center detection, membrane segmentation, and the correlation of OD on cells detected by algorithm vs cells annotated by pathologists are similar to a consensus of three pathologists.

	Metric	Average concordance between pathologists	IA vs pathologists
**A** QCS-IA: analytical results on development data, n=116 ROIs. Staining: Ventana 4B5 and research HER2 assay			
Epithelium detection	Dice scores	0.918	0.941
Cell center detection	F1 score	0.807	0.846
Membrane detection	Asymmetric surface distance	0.77 µm	1.13 µm
OD correlation on membrane	Pearson correlation	0.995	0.993
**B** Analytical results on J101 data, n=100 ROIs. Staining: HercepTest; Dako			
Epithelium detection	Dice scores	0.926	0.936
Cell center detection	F1 score	0.785	0.837
Membrane detection	Asymmetric surface distance	0.74 µm	1.08 µm
OD correlation on membrane	Pearson correlation	0.995	0.993

**C** TIL detection with IA: analytical results on development data, n=38 ROIs with pathologist annotations. Staining: Ventana 4B5 and research HER2 assay			
	Metric	Results	
TIL detection	F1 scores	0.72	
TIL detection	Spearman correlation on detected cell counts	0.93	

*HER2* human epidermal growth factor receptor 2, *IA* image analysis, *OD* optical density, *QCS* quantitative continuous scoring, *ROI* region of interest, *TIL* tumor-infiltrating lymphocyte.

The use of previously locked QCS-IA modules ensured unbiased IA of the cohort, which reflects a real-world scenario of unseen WSIs being received from a pathology laboratory for prospective analysis. Since our deep-learning–based QCS-IA modules were not trained on the HercepTest-stained images, there was a need to confirm the transferability of the solution to HercepTest-stained IHC images before application on the J101 data set. To confirm the performance on HercepTest-stained images, an analytical test was performed on the J101 images. Similar to the approach adopted for development data sets mentioned above, we benchmarked the IA results against ground truth annotations performed by a pool of three pathologists from J101 images. To this end, an additional 100 ROIs were selected, and each ROI was independently annotated by three pathologists. The IA analytical results are shown in Table 1B. This objective analytical evaluation showed remarkable generalization of deep-learning modules for epithelial region detection as well as subcellular segmentation, even though the QCS-IA modules were trained exclusively on Ventana 4B5-stained tissue sections without exposure to any HercepTest images. Further, we found that all the QCS-lA results were within the interobserver variance of the pathologists’ annotations.

For the analytical validation of TIL detection, an independent data set with 38 ROIs was annotated by one pathologist, who annotated 3122 TIL centers. The validation metrics chosen were the F1 scores to check the accuracy of TIL center detection. Additionally, we performed Spearman correlation analysis on the counts of detected vs annotated TIL centers. The results are summarized in Table 1C. In the absence of a functional lymphocyte marker (e.g. CD3), TIL detection is difficult. The annotations are based on the morphology of unstained cells, which are small, circular cells of uniform texture. F1 scores ≥ 0.7 are generally considered enough to provide robust trends on TIL densities. This was confirmed by the high observed Spearman correlation values (R = 0.93) of TILs counts detected by IA against a pathologist.

### QCS-based bioinformatics on J101—all available 151 BC samples

**Figure 3:**
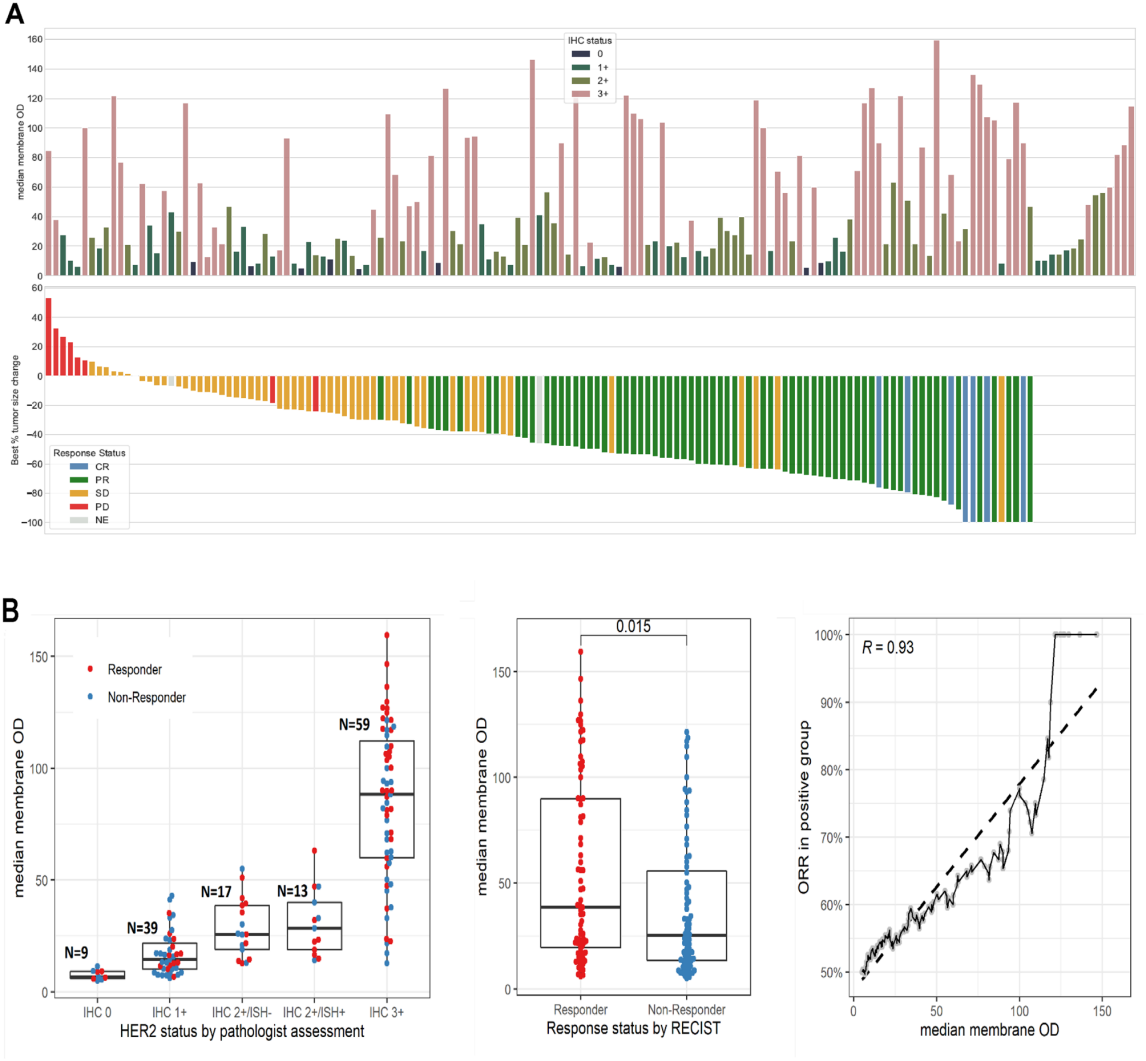
(**A**) In the bottom part of the figure, each bar represents a single patient with breast cancer in the NCT02564900 (DS8201-A-J101 [J101]) trial, with the bars sorted according to the best percentage change in tumor size, evaluated from baseline to on- or post-therapy follow–up examination by Response Evaluation Criteria in Solid Tumors (RECIST) version 1.1. The bars are color coded according to response status: complete response (CR), partial response (PR), stable disease (SD), or progressive disease (PD). Bars for non-evaluable (NE) response cases are not shown (in the bottom figure, on the right). In the top part of the figure, the bar height represents the median membrane optical density (OD) for all tumor cells per case as computed by quantitative continuous scoring (QCS). The bar color indicates the human epidermal growth factor receptor 2 (HER2) status as assessed by a pathologist. (**B**) Left: HER2 expression in J101 samples as measured by median membrane OD for each category of manual immunohistochemistry HER2 status determined by pathologists according to the American Society of Clinical Oncology/College of American Pathologists guidelines. Centre: Comparison of HER2 expression as measured by median membrane OD between responders and non-responders in J101. Responders are defined as CR and PR, and non-responders as SD, PD, and NE. Statistical significance was evaluated using the Wilcoxon test. Right: Pearson correlation between mean membrane OD and objective response rate in the QCS-positive group with the dashed line depicting the corresponding regression line. The QCS-positive group at a given OD value was defined as every patient with a mean membrane OD greater than that value. *IHC* immunohistochemistry, *ISH* in situ hybridization.

Once the QCS-IA was applied and OD-based measurements, such as mean OD per subcellular compartment, were obtained, we performed the bioinformatics part of the QCS pipeline. To begin with, we checked the correlation of HER2 IHC status as determined by pathologist scoring against the median membrane OD across all cells per sample (Fig. 3A). We noticed a trend toward higher median ODs with increasing IHC HER2 positivity but still observed an overlap between adjacent categories, which could be attributed to the HER2 pathologist scoring algorithm itself and the lack of its granularity and ability to provide continuous scores.

Correlation analysis of ORR in J101 against the median membrane OD across all cells per sample showed a significant difference in HER2 expression between responders and non-responders (Wilcoxon test *p*=0.015; Fig. 3B: Center). Furthermore, a direct monotonic relationship between ORR and increased HER2 expression across the entire range of the assay was observed (Pearson correlation: 0.93; Fig. 3B: Right). This suggests a continuous association of HER2 expression as determined by QCS with ORR in that each incremental increase in OD resulted in an increased response rate.

However, despite the statistical significance, there is still a large overlap in HER2 expression between responders and non-responders (Fig. 3B: Left). Thus, we aimed for an optimized model to better stratify patients using QCS-based features extracted from WSIs of HER2-stained tissue sections. To this end, a total of six features were generated for each patient: five features involving HER2 OD-based measurements and one stromal TIL (sTIL) density-based feature (see “Materials and methods”).

As each feature has a specific parameter set, a cross-validated grid search was used to identify the optimal combination of parameters and patient-level cut points for each feature. Evaluation of all parameters for all features resulted in a set of 269 readouts per WSI, although many of them were highly correlated. After readout consolidation (details in Materials and methods to remove non-informative and redundant readouts), we obtained 76 unique readouts that were then used for subsequent QCS-positive vs QCS-negative cut–point optimization. Stratification models were trained on the full data set, and the best-performing models were chosen based on log-rank *p*–value assessment between the QCS-positive and -negative groups in J101. To get an unbiased estimation of stratification performance of each readout on unseen data, a repeated cross-validation scheme was applied (see “Materials and methods”). During optimization, the QCS-negative population was constrained to be ≥20% of the total population. This constraint helps to avoid heavily unbalanced group sizes, which would diminish the statistical power of the log-rank test.

Table 2 lists the performance of different QCS-based features, the parameters, cut points, and the resulting log-rank *p* values, both from cross-validation as well as cut point optimization on the full training set (in brackets). Note that due to the exploratory nature of this analysis, we report unadjusted p values. While it is expected that cross-validated performance would be lower than the training-set performance, all top-ranked features still showed significant *p* values. This suggests generalizable features beyond the given training set. Generally, the best-performing QCS–based features were driven by a majority of tumor cells expressing a minimal amount of HER2. For instance, for OD quantiles (rank 3), an OD cut point of 7.21 was selected for the 5% quantile (i.e. for a patient to be considered biomarker positive by this model, 95% of tumor cells need to show a membrane OD of ≥7.21). Similarly, the percentage of membrane OD-positive tumor cells (rank 2) showed best stratification if >98.5% of all tumor cells were found at a membrane OD level of ≥6—an expression level at the edge of human perceptibility. This is in contrast to current clinical ASCO/CAP guidelines^2^, which suggest that efficacy is driven by a minority of cells expressing higher levels of HER2.

**Table 2:** (**A**) Results of PFS analysis using top-ranked HER2 QCS-based features.

Rank	Feature	Parameter	Cut point	PFS log-rank *p* value	Group	Prevalence	ORR
**A** Survival analysis from NCT02564900 (DS8201-A-J101 [J101]) data set (full cohort, N=151)							
Current standard: HER2 testing with ASCO/CAP guidelines							
ASCO/CAP HER2 score		IHC 3+/2+ ISH+		0.26	Positive	56.9	55.6
		IHC 2+ ISH-/IHC 1+/ IHC 0			Negative	43.1	41.5
Analysis with HER2 QCS-based features							
1	bSPS	R=50 µm, membrane OD ≥8	99.81	0.026 (0.00012)	High	76.4 [95% CI: 75.9–77.0] (Training: 77.5)	55.0 [95% CI: 54.6–55.5] (Training: 55.6)
					Low	23.6 [95% CI: 23.0–24.1] (Training: 22.5)	30.5 [95% CI: 29.5–31.4] (Training: 30.4)
2	% OD-positive cells	Membrane OD ≥6	98.51	0.033 (0.00017)	High	71.7 [95% CI: 71.2–72.3] (Training: 75.5)	54.5 [95% CI: 54.0–55.0] (Training: 55.3)
					Low	28.3 [95% CI: 27.7–28.2] (Training: 24.5)	35.7 [95% CI: 34.9–36.6] (Training: 32.4)
3	Membrane OD	5% quantile	7.21	0.026 (0.00054)	High	74.5 [95% CI: 73.9–75.0] (Training: 76.8)	54.6 [95% CI: 54.1–55.1] (Training: 55.2)
					Low	23.5 [95% CI: 25.0–26.1] (Training: 23.2)	33.3 [95% CI: 32.4–34.3] (Training: 31.4)
4	cSPS	R=25 µm, 5% quantile	37.18	0.014 (0.00077)	High	58.1 [95% CI: 57.7–58.6] (Training: 58.3)	57.3 [95% CI: 56.7–57.8] (Training: 58.0)
					Low	41.9 [95% CI: 41.4–42.3] (Training: 41.7)	38.9 [95% CI: 38.2–39.5] (Training: 38.1)
Analysis with TIL features							
sTILs		sTILs/mm^2^ inside tumor center	168.4	0.0070 (0.00055)	High	46.6 [95% CI: 46.1–47.0] (Training: 44.4)	49.2 [95% CI: 48.6–49.8] (Training: 49.3)
					Low	53.4 [95% CI: 53.0–53.9] (Training: 55.6)	50.2 [95% CI:49.7–50.8] (Training: 50.0)
**B** Survival analysis on NCT02564900 (DS8201-A-J101 [J101]) data set (HER2-negative subcohort, n=65)							
1	bSPS	R=50 µm, OD ≥8	99.81	0.0045	High	58.5	52.6
					Low	41.5	22.2
2	% OD-positive cells	OD ≥6	98.51	0.0072	High	57.9	48.6
					Low	42.1	21.4
3	STILs	sTILs/mm^2^ inside tumor center	168.4	0.36	High	43.0	39.2
					Low	57.0	43.2
**C** Survival analysis on J101 data set (HER2-positive subcohort, n=72)							
1	STILs	sTILs/mm^2^ inside tumor center	168.4	0.0009	High	43.0	58.0
					Low	57.0	53.6
2	% OD-positive cells	OD ≥6	98.51	0.029	High	90.2	56.9
					Low	9.8	42.8
3	bSPS	R=50 µm, OD ≥8	99.81	0.13	High	90.2	55.3
					Low	9.8	57.1

The feature cut points were optimized for the minimal, unadjusted log-rank *p* value within the given prevalence range. Unbiased performance estimates as derived via cross-validation are reported with training-set performance given in brackets. The features are ranked in order of increasing log-rank *p* values in the training set. The significance is reported at *p*≤0*.*05.

(**B**,**C**) HER2-negative and HER2-positive subgroup analysis using best HER2 QCS-based and TIL features. The signature cut points were optimized for the best PFS log-rank *p* value on the whole cohort and directly applied to the subcohorts. The features are ranked in order of increasing log-rank *p* values. Please note that from the full cohort of n = 151, 14 cases were classified IHC 2+ but have missing ISH status and thus cannot be unambiguously assigned to any subgroup.

% OD-positive cells, percentage of optical density-positive cells.

*ASCO* American Society of Clinical Oncology, *bSPS* binary spatial proximity scores, *CAP* College of American Pathologists, *CI* confidence interval, cSPS continuous spatial proximity score, *HER2* human epidermal growth factor receptor 2, *IHC* immunohistochemistry, *ISH* in situ hybridization, *OD* optical density, *OR*, *ORR* objective response rate, *PFS* progression-free survival, *QCS* quantitative continuous scoring, *sTIL* stromal tumor-infiltrating lymphocyte, *TIL* tumor-infiltrating lymphocyte.

**Figure 4:**
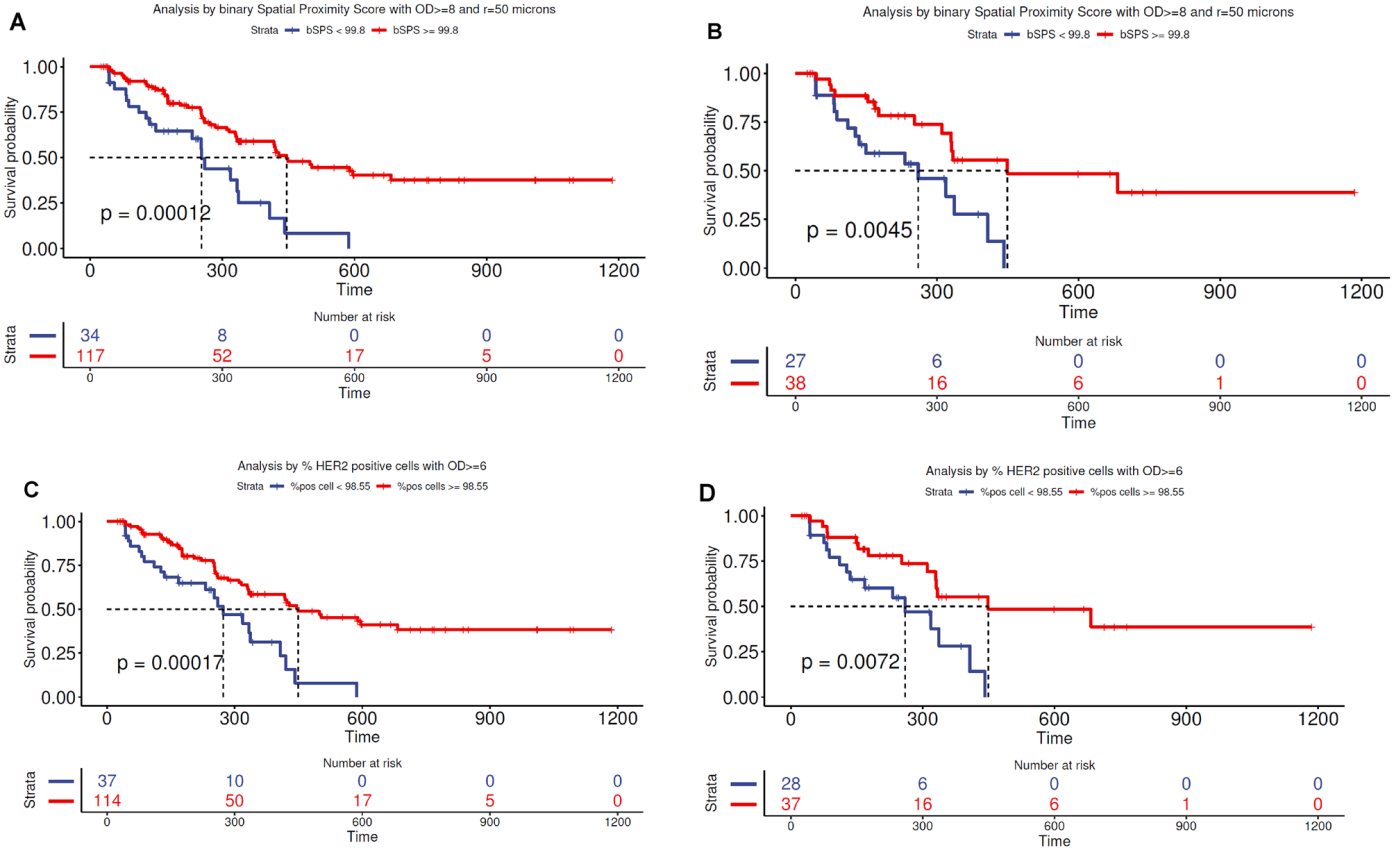
Progression-free survival in NCT02564900 (DS8201-A-J101) using the two best-ranked human epidermal growth factor receptor 2 (HER2) quantitative continuous scoring-based features. (**A**) Kaplan-Meier analysis with binary spatial proximity scores (bSPS), all patients. (**B**) Kaplan-Meier analysis with bSPS, HER2-negative subgroup. (**C**) Kaplan-Meier analysis with percentage of optical density (OD)-positive cells, all patients. (**D**) Kaplan-Meier analysis with percentage of OD-positive cells, HER2-negative subgroup.

**Figure 5:**
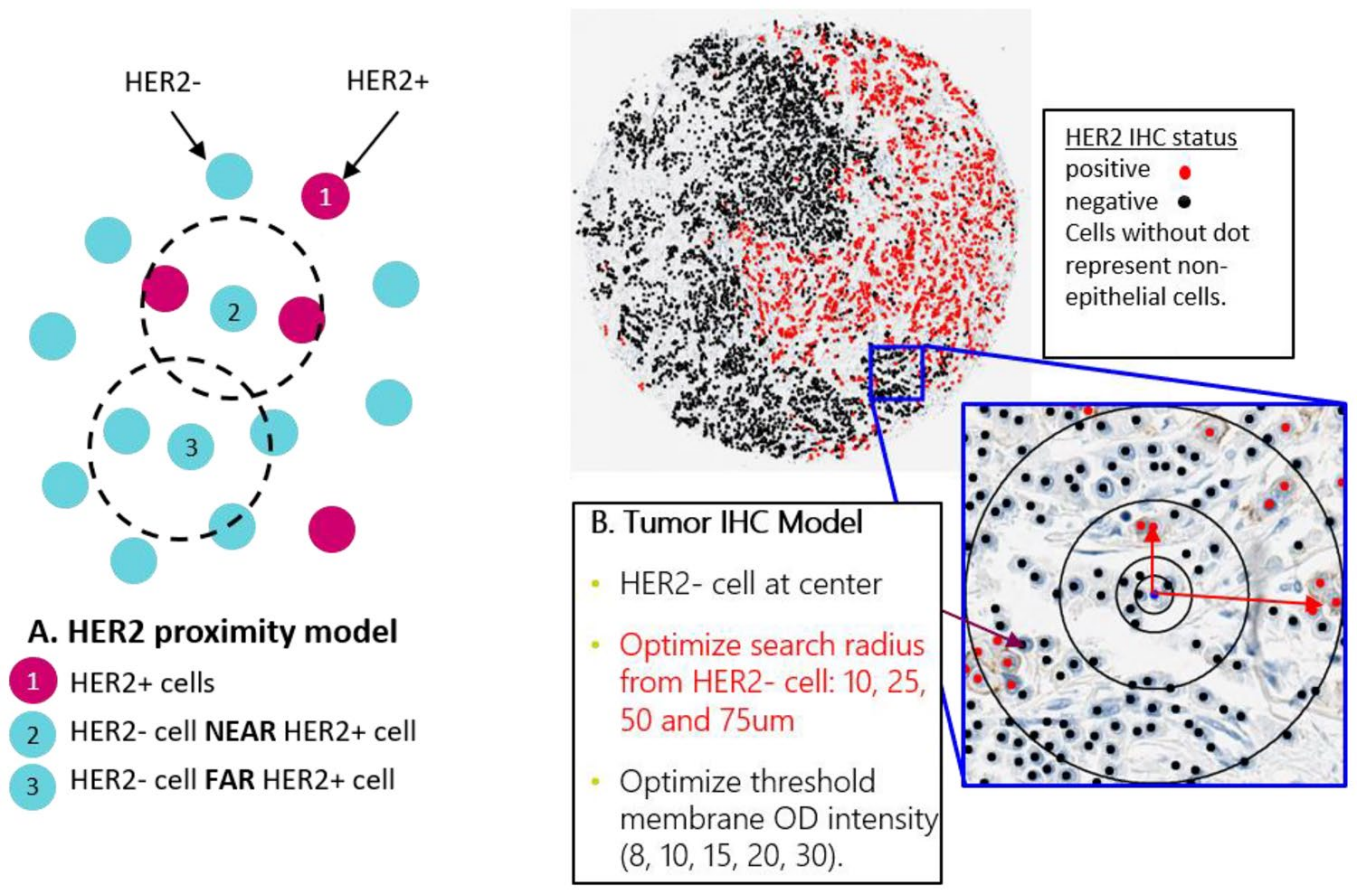
The spatial proximity score estimates potential bystander activity. The model takes into account the spatial location of the tumor cells as well as their human epidermal growth factor receptor 2 (HER2) expression in terms of optical density (OD) and computes the number of tumor cells that are likely to be killed as a result of diffusion of the antibody-drug conjugate payload subsequent to linker cleavage post internalization. Note that this model does not measure the bystander activity of the ADC itself. *IHC* immunohistochemistry.

The above-mentioned QCS-based features are based on aggregation of HER2 expression across the whole sample but ignore the rich spatial location information that QCS-IA–based detection offers, which helps to model spatial heterogeneity and potential bystander activity. To this end, we developed a novel scoring category called SPS. The binary version of SPS (bSPS) examines spatial heterogeneity by characterizing cells as showing a membrane OD above a determined OD threshold (OD-positive cell) or located within a certain distance from an OD-positive cell (for details, refer to Fig. 5 and Materials and methods). Patient stratification with bSPS (rank 1) yielded the best PFS log-rank *p* value on the full cohort (*p*=0.00012; Fig. 4A). Similar to the percentage of OD-positive cells (% OD-positive cells) (Fig. 4C), best-performing OD thresholds for bSPS, again, are found with a minimal level of HER2 expression (OD ≥8). The continuous version of SPS (cSPS; rank 4) results in significant stratification as well (*p*=0.00077; Table 2A). Of note, the cSPS gives the best ORR in the QCS-positive subgroup (58%), however, the performance of the model was lower compared with bSPS (58.1% prevalence vs 76.4% prevalence with bSPS).

For non-HER2 expression–related features, we discovered that the density of TILs in the tumor center (sTIL density) provides a significant stratification with cut points optimized on the full cohort (*p*=0.00055) as well as during cross-validation runs (see Supplementary Figs. S1 and S2). While sTIL density would rank fourth highest in terms of training-set *p* values, this feature showed the lowest cross-validated log–rank *p* value among all analyzed features.

### Analysis of HER2-negative population

In this study, we were particularly interested in the HER2-negative population as defined by standard clinical scoring (IHC 1+ or 2+/ISH−, n = 56 and HER2 IHC 0, n = 9). Despite the low HER2 score, a median PFS of 11 months was observed, and 40.0% of patients (26/65) in this subgroup in J101 responded to T-DXd treatment (Fig. 3A shows the waterfall plot of tumor shrinkage as related to QCS-membrane OD and IHC status). Thus, to confirm if the above observed stratification performance of HER2 QCS also retained the same value/score, we applied the best-performing models: % OD-positive cells, bSPS, and sTIL density, as obtained from the cut point optimization on the whole cohort, to the HER2-low population only. Results are summarized in Table 2B. Both the % OD-positive cells (PFS log-rank *p *= 0.0072) (Fig. 4D) and the bSPS (PFS log-rank *p *= 0.0045) (Fig. 4B) showed significant stratification for the HER2-low subset analysis and were able to identify 18 of 26 and 20 of 26 responders in the QCS-positive subgroup, respectively. sTIL densities were no longer able to separate long from short PFS (PFS log-rank *p*=0.36). In view of this clear difference between sTIL and HER2 QCS-based features that did not occur in the whole cohort but were visible in the HER2-low subgroup, we applied the same models to the HER2-positive subpopulation. This time, the sTIL densities were highly significant (PFS log-rank *p *= 0.0009) (Table 2C). In contrast, % OD-positive cells were left only weakly significant (*p *= 0.029), while bSPS showed no significance anymore (*p *= 0.13). Yet, when the very high prevalence of >90% for the QCS-positive groups of those two features is considered, this lack of significance is not surprising. In fact, this indicates that the HER2-positive population, according to ASCO/CAP guidelines, is almost entirely contained in the QCS-positive groups. However, QCS is able to extend this group into the otherwise HER2-negative population without sacrificing any stratification performance. This observation needs to be confirmed in an independent cohort.

## Discussion

The QCS-based survival analysis of the J101 study demonstrates that the use of digital QCS-based biomarkers could potentially enhance the prediction of patient response to T-DXd by increasing the sensitivity and specificity of the prediction, especially in the HER2-negative population. However, it is important to note that this is a purely exploratory study and therefore all of our findings would need independent validation to confirm any potential clinical utility.

QCS-based biomarkers extend the current standard scoring concepts that rely on estimating the percentage of positive cells by (a) providing a drug efficacy-driven definition of cellular positivity and (b) adding the spatial dimension with the SPS. The latter enables the assessment of intratumoral heterogeneity of HER2 expression. This phenotype is found in a subset of BCs, more commonly in BC with equivocal HER2 protein expression and low-grade *HER2* gene amplification, and is reported to be associated with poor clinical outcome in patients with HER2-positive primary BC. QCS-based biomarkers would benefit this population by selecting patients for T-DXd therapy with increased precision. It is important to note that bSPS is an extension of %OD positive cells (Refer to Eq. (3) in “Materials and Methods”), resulting in high overlap between biomarker positive and negative patients from the two features. The details in overlap of the signatures can be found in supplementary materials.

QCS-based analysis also models the role of TILs on patient survival. Although stratification based on the density of TILs in stroma provided an ORR of only 49%, the patients with high sTIL density show longer median PFS (see Supplementary Fig. S1). This suggests that, while not needed for an immediate response to T-DXd for a patient, sTILs play an important role in the time to progression. To what extent this is coupled to a generally prognostic effect of TILs in this setting vs a predictive effect specific to T-DXd is to be confirmed in a further study. On that note, to confirm the significance of all QCS-based biomarkers found in this study, an independent validation cohort will be needed. In that cohort, we would apply a locked version of QCS-based analysis for which we would apply the locked IA and scoring parameters (e.g., OD thresholds, SPS radius) identified in this study for patient stratification. It is important to note that the clinical trial setting of other cohorts in terms of assay, sample processing, and imaging protocols, including tissue sectioning thickness and scanner color calibration, should be similar to those of the J101 cohort analyzed in this study. When other clinical trial cohorts are stained with different assays (e.g. Ventana 4B5), the cell-level OD thresholds and patient-level QCS cut points obtained with HercepTest staining might not be directly applicable to those cohorts.

J101 was a single-arm study with no standard-of-care control arm (treatment of patients with chemotherapy and/or radiation therapy). Future clinical trials with a standard-of-care arm will help us gauge the predictive capability of the signatures found in this study. Also, the J101 data set was skewed toward HER2-positive patients, with a low number of HER2-negative patients, which does not reflect real-world BC epidemiology. The current system does not consider the circumferentiality and completeness of the membrane in any of the scores. Current ASCO/CAP guidelines^2^ use circumferential membrane staining in addition to staining intensity to discriminate HER2 IHC 1+, 2+, and 3+ cases. Future extensions of this work might look at including these features in QCS. Last, the J101 follow-up clinical data is too immature at this time of analysis to include QCS optimization with respect to overall survival. While we recognize the limitation of this study to assess heterogeneity of metastatic expression and its confounding links to ORR/PFS, the robust enrichment of ORR/PFS with QCS suggests the superiority of QCS features for predicting outcome.

We envision that similar algorithms can be developed for other tumor types (e.g. lung and gastric), which aim to help extend the patient population benefiting from T-DXd treatment. More generally, QCS-based analysis can be performed using any digitized IHC slide stained with a membrane-specific marker. QCS-based analysis provides flexibility to tailor scoring schemes to different MOAs of the investigated drugs, which provides a potential opportunity for wide applications of QCS-based biomarker analysis. To support this, we performed a successful QCS-based analysis on a data set of durvalumab-treated (anti–programmed death-ligand 1 [PD-L1]) patients with late-stage non–small cell lung cancer^11^ (see Supplementary Figs. S3, S4, S5).

For deployment of such a digital pathology system to the clinics, we will need to standardize the tissue preparation protocols (e.g. section thickness), IHC staining, and slide digitization. Current ASCO/CAP guidelines^2^ provide recommendations regarding tissue fixation and quality. In this study, the section thickness directly impacted the OD values. In the J101 analysis, all samples were reprocessed in a central laboratory and hence followed the same protocol. However, in a real-world setting with decentralized testing, with the samples processed in different laboratories, section thickness and differences in staining protocols might be an issue. These issues can potentially be resolved using normalization of OD values against standardized values on slide controls.

Another planned extension of the current work is to further improve the epithelium detection module to detect invasive cancer and separate out the in-situ (ductal/lobular) and non-cancerous epithelium with a deep-learning model. This will avoid any human intervention and any source of human subjectivity in the system and, hence, will make the system fully automated.

## Materials and methods

QCS is a two-part process. The first part comprises IA components to quantify HER2 expression on tumor cells using a continuous scale (Fig. 1B). The second part is the bioinformatics part, in which we analyze the QCS-IA data against clinical variables. The IA involves inputting a digitized WSI acquired from a tissue section stained with an approved HER2 assay (e.g. Ventana 4B5, HercepTest [Dako]). Within the bioinformatics part, the cell-level information is aggregated to slide-level information (e.g. as a histogram of frequency of cells by OD). Together with the spatial information encoded by the center of gravity of each cell within the WSI, novel biomarkers are derived by correlating these single-cell features to clinical response and survival data.

### QCS-image analysis approach

We developed a deep-learning–based instance segmentation method to segment subcellular structures in WSIs acquired from IHC HER2-stained BC tissue sections using the dye DAB and hematoxylin as a counterstain. The method comprises two deep-learning models that work in cascade: the first model detects the epithelial regions, and the second model detects the cell instances and segments the subcellular compartments (i.e. the membrane, nucleus, and cytoplasm for each detected cell within the epithelial regions). Fig. 1B shows the overview of the QCS-IA workflow for epithelial, single cell, and subcellular structure detection. While there are other one-step instance segmentation methods in the literature^12^, this two-step approach was chosen to ensure model modularity and reusability. For instance, the cell detection model can be reused for subcellular segmentation for other ADC or non-ADC targets (refer to the Supplementary Information for the application of QCS-IA on PD-L1 images by coupling the subcellular segmentation model with the PD-L1 epithelial detection model). All the cells detected outside the epithelial regions are excluded automatically. Note that non-neoplastic epithelium regions, as well as in-situ carcinomas (ductal and lobular), were also detected as epithelial regions by the model because it was developed to detect all kinds of epithelium. Thus, before the WSI analysis, an expert provided detailed tumor core annotations to avoid analysis on non-neoplastic epithelium and in-situ carcinoma regions. Within the tumor-epithelial region, the cell instances were segmented using a seeded watershed algorithm with cell centers as seed points and membrane posteriors as energy to stop the region growing process. The membranes are restricted heuristically to a 2 to 4-pixel thickness. Nuclei instances are segmented using thresholding of the nuclei posterior maps, where pixel values ≥0*.*5 are considered as nuclei. The remaining area of the cell after nuclei and membrane segmentation is assigned as cytoplasm. Fully supervised deep learning was used to train both models solely based on data annotated by experts.

#### Development data sets

The models were trained on BC WSIs obtained from different bio-bank sources. The acquired BC samples included resections (n = 85) as well as core needle biopsies (n = 40), and tissue microarrays (n = 100 cores) stained with Ventana PATHWAY 4B5 as well as with a research-use only HER2 assay. To ensure model generalization across different assays, staining patterns (faint, weak, moderate, intense), different tissue types (resections, biopsies, tissue microarrays), and morphologies (ductal, lobular) in ROIs were carefully chosen to be annotated for training and model selection (model validation). The annotations on the chosen ROIs were performed by pathologists or biomedical experts under pathologist supervision. No data from clinical trials (neither images nor clinical information) were used in the development of the QCS-IA models.

For the epithelium detection model, 2157 ROIs were annotated with sizes varying from 200 to 500 µm in diameter. The epithelial regions within these ROIs were precisely delineated by experts. In addition, all non-epithelial regions (stroma, artifacts like tissue folds, or scanning blur) within and outside of epithelial regions were delineated to ensure that the model learned to precisely detect epithelium regions and automatically reject all other regions for analysis.

For the cell segmentation model, a subset of 356 ROIs, as described above, were annotated for cell centers, nucleus outlines, and membrane outlines. We used a seeded watershed algorithm using annotated cell centers as a seed, and a brownness layer as an energy map to propose cell boundary candidates. In case the watershed algorithm did not give correct membrane outlines, they were manually corrected by experts. This semi-automated process proved to be an efficient way of collecting large numbers of precise membrane annotations.

For the TIL detection model, a separate subset of 376 ROIs was annotated for TILs. Point annotations were used to indicate TILs, which, in the absence of functional staining, are characterized as round to polygonal, relatively small cells with little cytoplasm and a nucleus with homogeneous texture. In total, approximately 55,000 TIL annotations were obtained. In this work, we focused on sTILs as well as intraepithelial TILs.

#### Algorithm training, testing, and model lock

For all three models (epithelium detection, cell segmentation, TIL detection), a UNet^13^ with a ResNet50^14^ backbone was trained using the Adam optimizer (LR = 0.001, beta1 = 0.5, beta2 = 0.999) on a single Nvidia V100 GPU. The model parameters were chosen based on the best performance on a validation set. The training and validation sets were split into roughly 70% and 30% subsets, respectively.

#### QCS-image analysis of J101 data set

The J101 BC tissue samples (N = 151; described in Fig. 2) were stained with HercepTest antibody, and the WSIs were digitized using an Aperio scanner at 20× magnification. All the WSIs underwent a strict quality check by experts to ensure acceptable scanning quality, tissue preparation, and presence of enough viable tumor cells (*>*100 tumor cells). After the quality check, all 151 samples remained where the analysis was performed. The IA module was blindly applied on the J101 data (i.e. no samples from the J101 data set were used to develop the IA module). The blind application of existing IA models ensured unbiased analysis of the cohort to mimic a potential real-world scenario of unseen samples coming in for analysis from a laboratory.

### QCS-bioinformatics approach

With the help of QCS-IA, subcellular compartments for each tumor cell per WSI were precisely segmented, and OD values for “brownness”^15^ on the membrane and cytoplasmic subcompartments were computed as the mean OD across all pixels of respective subcompartments (see Supplementary Fig. S6). This approximates the amount of DAB precipitate and, in turn, the level of HER2 protein expression. The OD estimation in HER2 stained chromogenic IHC images has shown to correlate well with corresponding color-deconvoluted multiplexed immunofluorescence data and transcriptomics data in serial sections^16^. Based on the distribution of OD values in both compartments and the spatial location information of tumor cells, five different categories of HER2 QCS-based features were derived. Each category represents a specific property of target expression on the tumor cell level. To get from a cell level value to a patient level score, a specific aggregation function is applied that usually needs one or more parameters to be specified. Finally, to get a fully defined biomarker candidate that can be used for patient stratification on the cohort level, a cut point is needed to separate patient populations that can be called biomarker-high and biomarker-low, respectively. The following two subsections describe the five classes of cell level features and how they are aggregated to patient level scores including a definition of the required parameters. This is followed by a subsection describing how these scores are used to derive cohort level cut points for patient stratification.

#### Basic HER2 QCS readouts

The basic QCS-based readouts are derived from aggregation of HER2 OD values from cell level to case level. A specific set of parameters was computed from histograms such as OD quantiles, % OD-positive cells (cells having an OD larger than a predefined threshold), and average number of OD-positive cells in tumor epithelium per mm^2^ (OD-positive cell density).

- OD levels. Different quantiles were derived from the membrane OD distribution starting from the fifth up to the 95th percentile in steps of 5%. In addition, mean OD per case was evaluated.
- Percentage of OD-positive tumor cells. The percentage of tumor cells per case above a given OD threshold was recorded for various thresholds.
  1 $$\%positive \,\,tumor \,\,cells\left(O{D}_{threshold}\right)= \frac{{\sum }_{i}{\mathbb{I}}[O{D}_{membran{e}^{i}}\ge O{D}_{threshold}]}{N}.$$

To define positive cells, a list of preselected OD thresholds (6, 8, 10, 12, 15, 20, 25, 30, 40, 50, 60, 70, 80, 90, 100, 110, 120) was explored. These thresholds were chosen by the perception of the human eye, from 6 being very faint to 120 being very strong brown staining. Note that for the purpose of this study, we focused on the lower end of the HER2 expression range.

- Density of OD-positive epithelial cells. Similar to the percentage of positive cells, this type of readout requires a predefined OD threshold to discriminate positive from negative cells.
  2 $$density(O{D}_{threshold)}= \frac{{\sum }_{i}{\mathbb{I}}\left[O{D}_{membran{e}^{i}}\ge O{D}_{threshold}\right]}{are{a}_{epithelium}}.$$

The same list of OD thresholds as described above was used. However, densities are calculated relative to the size of the analyzed area, resulting in the number of OD-positive cells per mm^2^.

#### Spatial proximity scores

Ogitani et al.^17^ suggest that T-DXd treatment exhibits bystander activity (i.e. not only is a HER2-positive cell likely to be killed, but the neighboring cells around the HER2-positive cells are also likely to be killed). With the data generated from QCS-IA (cell coordinates, cell membrane OD), we propose a novel mathematical model for analysis of spatial heterogeneity to understand the potential bystander activity and its impact on patient survival. Figure 5 shows a schematic of the bSPS.

The bSPS model extends the % OD-positive cells model by incorporating neighborhood information.3

N refers to the total number of detected tumor cells on the slide; N_i_^r^ refers to all the cells in the neighborhood of a cell *I* within a radius of *r*. The *OD*_*threshold*_ refers to the OD threshold set to label a cell OD positive. The model estimates the likelihood of a tumor cell to be killed by the drug. It is based on two conditions: (a) the tumor cell itself is OD positive or (b) any neighboring cell within a radius *r* is OD positive. Once the total number of cells likely to be killed is determined, the bSPS is defined as the fraction of such cells among all tumor cells. The bSPS is a more generalized form of % OD-positive cells. In Eq. (3), with *r* → 0, the neighborhood reduces to the cell itself and becomes Eq. (1).

bSPS applies binary thresholds to decide upon OD positivity (any cell with a membrane OD value above *OD*_*threshold*_ is considered positive, independent of the actual OD level) and the weighting of neighboring cells (any neighbor within a radius *r* is considered equally, no matter the actual distance to cell *i*). Also, the total number of OD-positive neighbors is not reflected in this score; a single positive neighbor is counted exactly the same as 10 or 100 positive neighbors would be. To account for these limitations, a second model called the cSPS was defined as a generalization of the OD levels per cell. This yields a continuous value for the OD positivity in the neighborhood of a cell *i*:4*d*_*i j*_ is the distance from cell *i* to its neighbor *j*. Basically, cSPS is a weighted sum of the OD values within the whole neighborhood *N*_*i*_^*r*^, using a linearly decreasing weighting function based on the distance between cells *i* and *j*. Similar to cellular OD levels above, cSPS values were aggregated using the mean and several quantiles across each case to generate a set of final scores for further analysis. In this study, we used all combinations of *r* ∈ (10, 25, 50, 75) µm together with the mean and all quantiles from the fifth to the 95th percentile in steps of 5%. Again, with *r* → 0 Eq. (4) reduces to the above-described OD levels of individual cells.

Further explanation of all QCS features is also available in Supplementary Table S2.

#### Cut point optimization

The full set of generated WSI readouts, as described above, were filtered to remove any non-informative and redundant readouts. Any readout comprising zero or near-zero variance was considered non-informative. This was defined as the readouts with only one unique value (zero variance) or features that have both of the following characteristics (near-zero variance): the number of unique values divided by the number of samples is <0.1, and the ratio of the frequency of the most common value to the frequency of the second most common value is >95:5. Next, to remove redundant features, all pairwise Spearman correlation coefficients were calculated. From each pair with a correlation of ≥0.99, the readout with the larger mean absolute correlation across the whole readout set was discarded. Mean absolute correlations were re-evaluated at each step.

After readout consolidation, systematic cut point optimization was applied to all remaining readouts to derive accurate univariate models for patient stratification. Therefore, each data point in the training set was considered a potential binary cut point to separate patients into two groups. All cases with readout values greater than or equal to the current cut point were assigned to the QCS-positive group, while cases with readout values lower than the cut point were assigned to the QCS-negative group. Two independent target functions were used to identify optimal cut points: first, we maximized ORR in the positive group to stratify responders (complete and partial response) vs non-responders (stable disease, progressive disease, non-evaluable). Second, we minimized PFS log-rank *p* values for the separation of QCS-negative and -positive subgroups. In both cases, the prevalence of both negative and positive was constrained to be ≥20%.

To increase the robustness of the trained cut points, a bootstrap approach was applied. Fifty random bootstrap samples were drawn from the training set, and optimal cut points were derived for all of them. For each optimization target, the most frequent cut point among all bootstrap samples was then recorded as the optimal cut point for the full training set.

#### Repeated cross-validation

In this study, any analysis was restricted to a single available data set (J101) of T-DXd–treated patients. However, optimizing stratification cut points and evaluating their performance on the same data set bears the risk of overfitting. Thus, to increase our confidence in the robustness of the selected features for patient stratification, we applied an *n*-times repeated *k*-fold cross–validation scheme during cut point optimization. In this setting, the full data set was repeatedly partitioned into *k* equally sized random subsets, resulting in *n* different random splits. For a given split, all but one of the *k* subsets were used for training an optimal cut point per feature, as described above. These cut points were then applied to the remaining unseen subset to test stratification performance. This procedure was repeated for all *n* random splits. Our performance metrics comprise the prevalence of the high-scoring group, the ORR in both groups, and the log-rank statistic using PFS data. Prevalence and ORR values were averaged across all *n*-times *k* test folds evaluated during cross-validation. To evaluate log-rank performance, high- and low-scoring patients were pooled across all *k* test folds within one split to derive cross-validated Kaplan-Meier curves. However, standard log-rank tests would not be valid on such curves since cases grouped across folds would no longer be independent observations^18^. Instead, to be able to evaluate the statistical significance of the Kaplan-Meier curves, a permutation test was applied to derive *empirical p* values using *m* random permutations of the PFS and response information. The above-described *k*-fold cross–validation was then applied to each of the permutations to derive the distribution of the log-rank statistic under the null hypothesis of independence of considered features and outcome. In this study, cross-validation parameters were chosen as *k *= 5, *n *= 300, *m *= 1200.

### Ethics declaration

The clinical trial (J101) has been described previously^9^. The study was approved by the institutional review board at each site and conducted in adherence with the International Council for Harmonisation Good Clinical Practice guidelines, the Declaration of Helsinki, and local regulations on the conduct of clinical research. All patients provided written informed consent before participation in the trial.

### Supplementary Information

Supplementary Information.

## Data Availability

The data sets generated and/or analyzed during the current study are not publicly available owing to ongoing work on the data analysis. Please contact Danielle Carroll (danielle.carroll@astrazeneca.com) or Zenta Tsuchihashi (ztsuchihas1@dsi.com) for any data requests relating to this study.

## Code availability

Source code is proprietary information of AstraZeneca and will not be made publicly available.
