# Supplementary material for: HER2 quantitative continuous scoring for accurate patient selection in HER2 negative trastuzumab deruxtecan treated breast cancer
Source: Sci Rep. 2024 May 27;14:12129. doi: 10.1038/s41598-024-61957-9 (PMC11130140; doi:10.1038/s41598-024-61957-9)
Supplement: Supplementary file 1 — Supplementary Information. [file 41598_2024_61957_MOESM1_ESM.docx]

# Supplementary Information

HER2 quantitative continuous scoring for accurate patient selection in
HER2-negative trastuzumab deruxtecan–treated breast cancer

Ansh Kapil^1^, Andreas Spitzmüller^1^, Nicolas Brieu^1^, Susanne Haneder^1^,
Anatoliy Shumilov^1^, Armin Meier^1^, Fabiola Cecchi^2^, Alice Barkell^3^, Nathalie Harder^1^, Katrin Mittermaier^1^, Ana Hidalgo-Sastre^1^, Regina Alleze^1^, Markus Schick^1^,
Günter Schmidt^1^, Hadassah Sade^1^, Zenta Tsuchihashi^4^, Fumitaka Suto^4^,
Mark Gustavson^2^, J. Carl Barrett^2^ & Danielle Carroll^3^

^1^Computational Pathology, Oncology R&D, AstraZeneca, Munich, Germany ^2^Translational Medicine, Oncology R&D, AstraZeneca, Gaithersburg, MD, USA ^3^Translational Medicine, Oncology R&D, AstraZeneca, Cambridge, UK

^4^Translational Science, Daiichi Sankyo, Inc., Basking Ridge, NJ, USA

**Supplementary Table S1.** Terms and definitions used in this manuscript.

| **Term** | **Definition** | **Reference** |
| --- | --- | --- |
| HER2 IHC status 3+ | Circumferential membrane staining that is complete, intense, and in *>*10% of tumor cells | 2018 ASCO/CAP^1^ |
| HER2 IHC status 2+ | Weak to moderate complete membrane staining observed in *>*10% of tumor cells | 2018 ASCO/CAP^1^ |
| HER2 IHC status 1+ | Incomplete membrane staining that is faint or barely perceptible and within *>*10% of the invasive tumor cells | 2018 ASCO/CAP^1^ |
| HER2 IHC status 0 | No staining observed or incomplete membrane staining that is faint or barely perceptible and within ≤10% of the invasive tumor cells | 2018 ASCO/CAP^1^ |
| HER2-positive case | HER2 IHC status 3+ or HER2 IHC status 2+ and ISH+ | 2018 ASCO/CAP^1^ |
| HER2-low case | HER2 IHC status 1+ or HER2 IHC status 2+ and ISH− | Miglietta et al.^2^ |
| HER2 QCS score | Statistical aggregation of OD and spatial properties of tumor cells in a WSI. Common aggregation methods involve computation of quantiles of the distribution of OD values of tumor cells or of the  % OD-positive tumor cells |  |
| HER2 QCS-positive  case | HER2 QCS score of a WSI is larger than a predefined cut point |  |
| Membrane OD | Estimation of the amount of DAB and, therefore, HER2 protein on tumor cell membrane using the HSD algorithm for digital WSIs |  |
| OD-positive cell | Tumor cells that shows an average membrane and/or cytoplasm OD larger than a predefined threshold |  |

ASCO, American Society of Clinical Oncology; CAP, College of American Pathologists; DAB, 3,3'-diaminobenzidine; HER2, human epidermal growth factor receptor 2; HSD, hue saturation density; IHC, immunohistochemistry; ISH, in situ hybridization; OD, optical density; QCS, quantitative continuous scoring; WSI, whole-slide image.

**Supplementary Table S2.** Explanation and details of QCS features used in J101 study.

| **Feature** | **Explanation** |
| --- | --- |
| Membrane OD levels | Measure of **staining intensity** at membrane of tumor cells. E.g., 10% quantile means that 10% of tumor cells in a given case show an OD level below the reported value while the remaining 90% show a higher OD level. |
| % OD-positive cells | **Measure of the abundance of positive cells** at a given intensity level relative to the total number of epithelial cells:  *# positive tumor cells/# all tumor cells*  OD threshold defines the minimal OD level for a cell to be considered positive. |
| Density of OD positive cells | Measure of the abundance of positive cells at a given intensity level relative to the area size of the region of interest (ROI):  # positive cells/mm2  OD threshold defines the minimal OD level for a cell to be considered positive. ROI can be tumor epithelium only or whole tumor region (including tumor associated stroma). |
| Binary Spatial Proximity Score (bSPS) | The percentage of tumor cells that are likely to be affected either directly (due to their own target expression) or via bystander activity (due to any neighbor’s target expression). The radius defines the maximum distance to consider a positive neighbor, the OD threshold defines the minimum OD to consider a cell positive. |
| Continuous Spatial Proximity Scores (cSPS) | The total OD level contributing to a tumor cell’s susceptibility to a given drug due to their own target expression or their neighbors’ target expression (potential bystander activity). Neighbor contributions are weighted by their distance. The radius defines the maximum distance to consider a neighbor. E.g., 25% quantile means that 25% of tumor cells in a given case show a total OD level below the reported value while the remaining 75% show a higher value. |

OD, optical density; QCS, quantitative continuous scoring; WSI, whole-slide image.

**Supplementary Table S3.** Explanation, illustration, and details of image analysis testing metrics.

| **Test** | **Metric** | **Illustration** | **Details** |
| --- | --- | --- | --- |
| Epithelium detection | Dice Score | 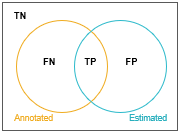 | The dice score measures the overlap between annotated (GT from pathologists) and the estimated (or predicted by QCS model) regions. For regions, true positives (TP), false positives (FP), false negatives (FN) and true negatives (TN) are defined by overlaps as suggested in the illustration.  **Dice Score = 2 TP / (2 TP + FP + FN)** |
| Cell center detection | F1 Score | 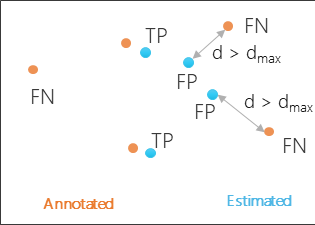 | For cell centers (both QCS and TIL models), the TP detection is defined when the estimated (prediction) and the GT annotations are within a distance d_max. In our experiments, d_max is chosen to be 5 micrometers [Brieu, SPIE, 2017]. On the same lines FP (when a predicted cell cannot be matched to GT cell) and FN (when there is no predicted cell at a location where GT is available).  **F1 Score = 2 TP / (2 TP + FP + FN)** |
| Membrane detection | Asymmetric surface distance | 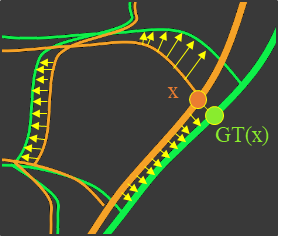 | The asymmetric surface distance^6^ is a measurement of the average distance of the surface of a segmented object (x) and the ground truth (GT(x)), defined as boundary test annotations drawn by trained pathologists. |
| OD correlation on membrane | Pearson correlation | 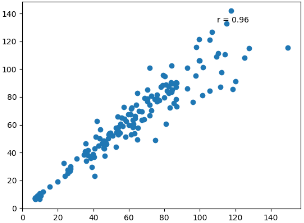 | The Pearson correlation is used to measure the strength of the linear relationship between the membrane OD and when segmentation is done by QCS vs OD with pathologist annotated membranes. |

OD, optical density; QCS, quantitative continuous scoring; GT, ground truth; TP, true positives; FP, false positives; TN, true negatives; FN, false negatives; TIL, tumor infiltrating lymphocytes.

# Supplementary Table S4. Results of PFS Kaplan-Meier analysis using bivariate combinations of top-ranked HER2 QCS–based features and cell densities of stromal TILs. Features are combined using a simple logical OR condition. Thus, for patients to be considered positive for the combination signature, it is sufficient if they are positive for >1 of the previously identified individual features.

| **Rank** | **Feature** | **Parameter** | **Cut point** | **PFS log-rank  *p* value** | **Group** | **Prevalence** | **ORR** |
| --- | --- | --- | --- | --- | --- | --- | --- |
| PFS Kaplan-Meier analysis on J101 data set with combination features (full cohort, N=151) | | | | | | | |
| 1 | bSPS or sTILs | bSPS ≥99.81 or density ≥168.4 sTILs/mm^2^ | | 0.00008 | High | 86.7 | 51.9 |
|  |  |  |  |  | Low | 13.3 | 35.0 |
| 2 | % OD-positive cells or sTILs | % OD-positive cells ≥98.51 or density ≥168.4 sTILs/mm^2^ | | 0.00028 | High | 86.7 | 51.9 |
|  |  |  |  |  | Low | 13.3 | 35.0 |

% OD-positive cells, percentage of optical density-positive cells; bSPS, binary spatial proximity scores; HER2, human epidermal growth factor receptor 2; OD, optical density; ORR, objective response rate; PFS, progression-free survival; QCS, quantitative continuous scoring; sTIL, stromal tumor-infiltrating lymphocyte; TIL, tumor-infiltrating lymphocyte.

# Additional Kaplan-Meier analysis curves for progression-free survival with data from NCT02564900 (DS8201-A-J101 [J101])

**Supplementary Figure S1.** Kaplan-Meier curves for patient stratification on J101 data set using the stromal TIL densities on (**A**) the full cohort, (**B**) the HER2-negative cohort, and (**C**) the HER2-positive cohort. HER2, human epidermal growth factor receptor 2; sTIL, stromal tumor-infiltrating lymphocyte; TIL, tumor-infiltrating lymphocyte.

**A
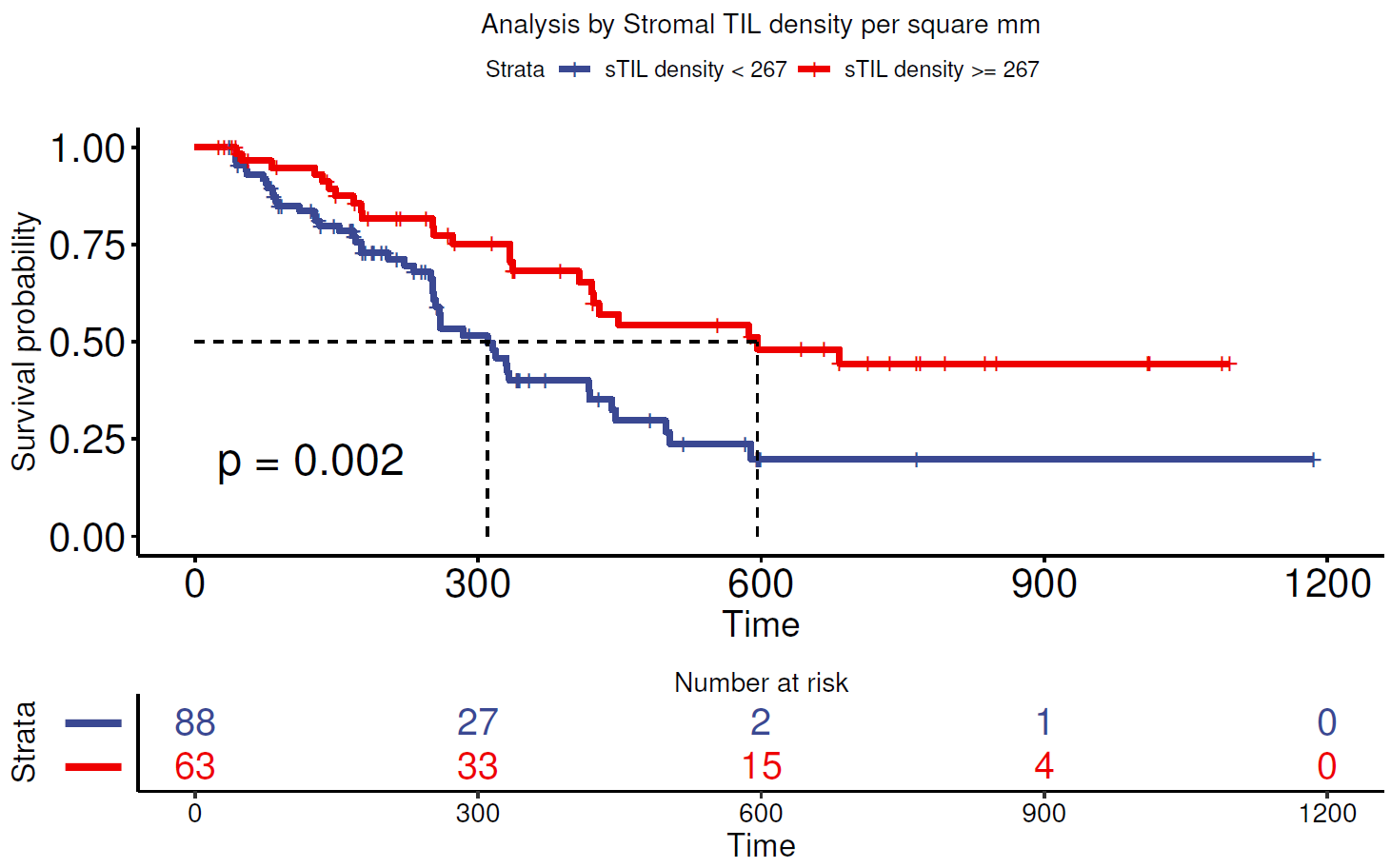
**

**B**

**
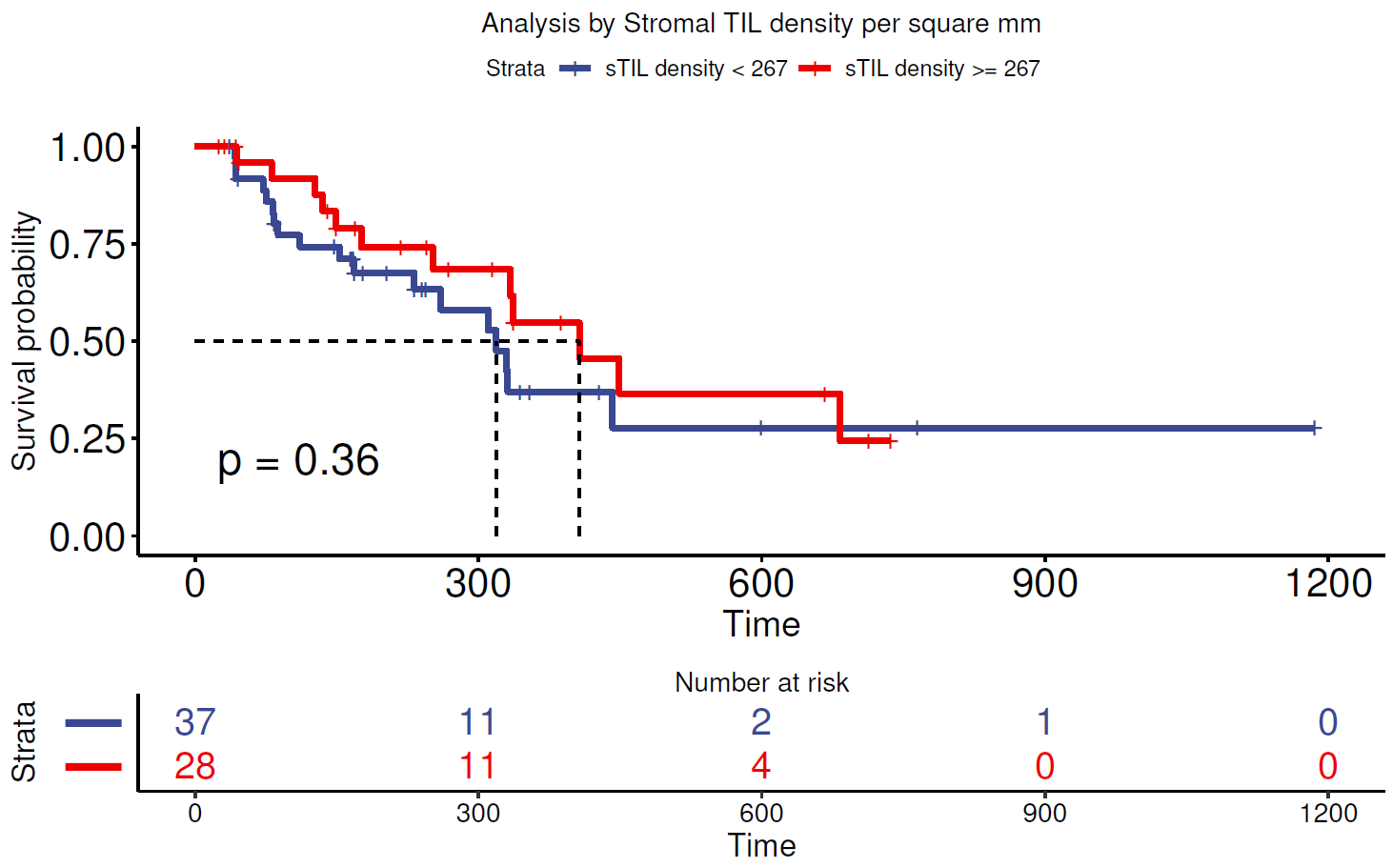
**

**C**

**
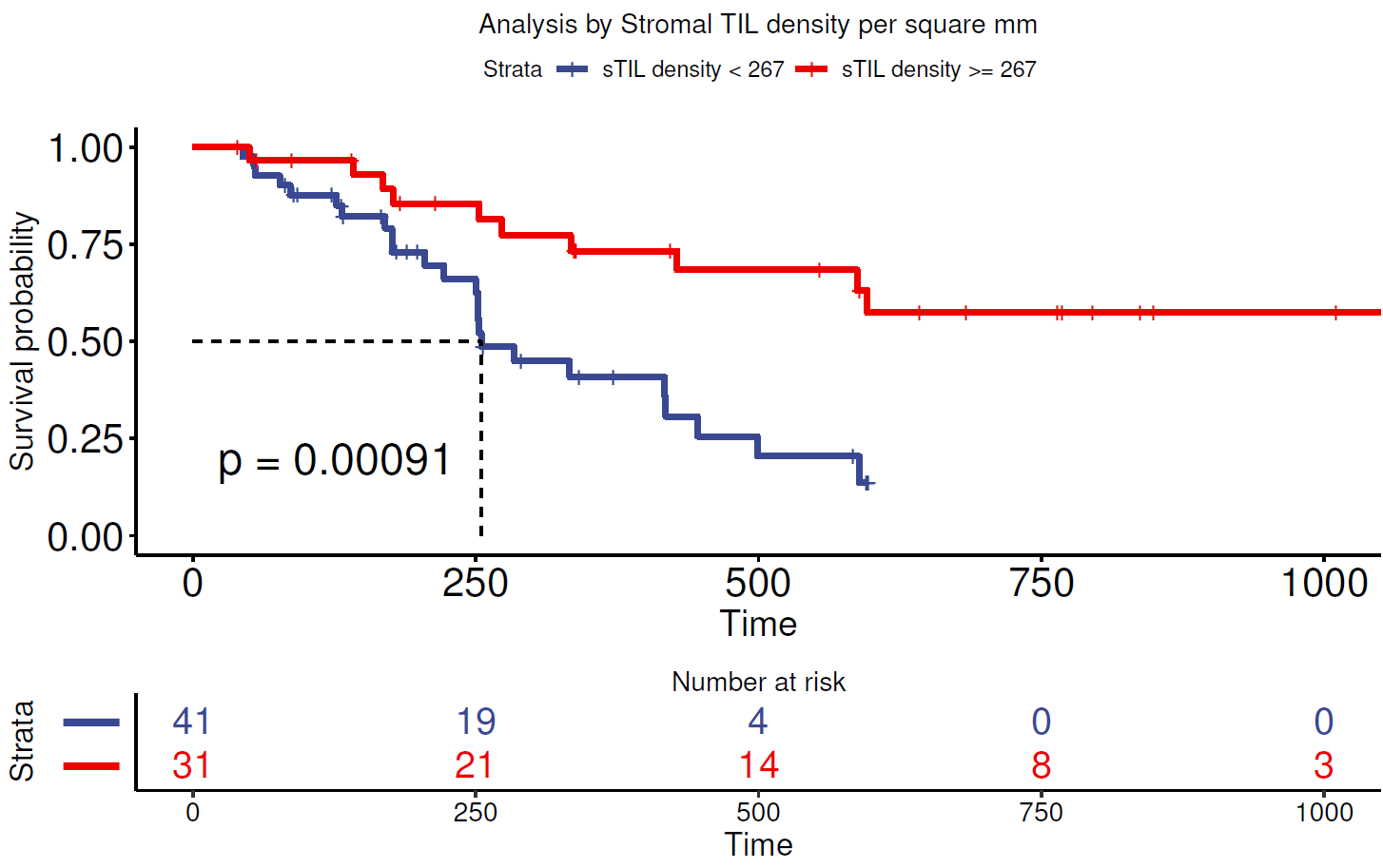
**

**Supplementary Figure S2.** Kaplan-Meier curves for patient stratification on J101 data set using the bivariate combination of best HER2 QCS-based features and sTIL densities. Stratification using % OD-positive cells or sTIL density for (**A**) the full cohort, (**B**) the HER2-negative cohort, and (**C**) the HER2-positive cohort. Stratification using bSPS or sTIL density for (**D**) the full cohort, (**E**) the HER2-negative cohort, and (**F**) the HER2-positive cohort. bSPS, binary spatial proximity scores; HER2, human epidermal growth factor receptor 2; Neg, negative; OD, optical density; Pos, positive;
QCS, quantitative continuous scoring; sTIL, stromal tumor-infiltrating lymphocyte.

**A**
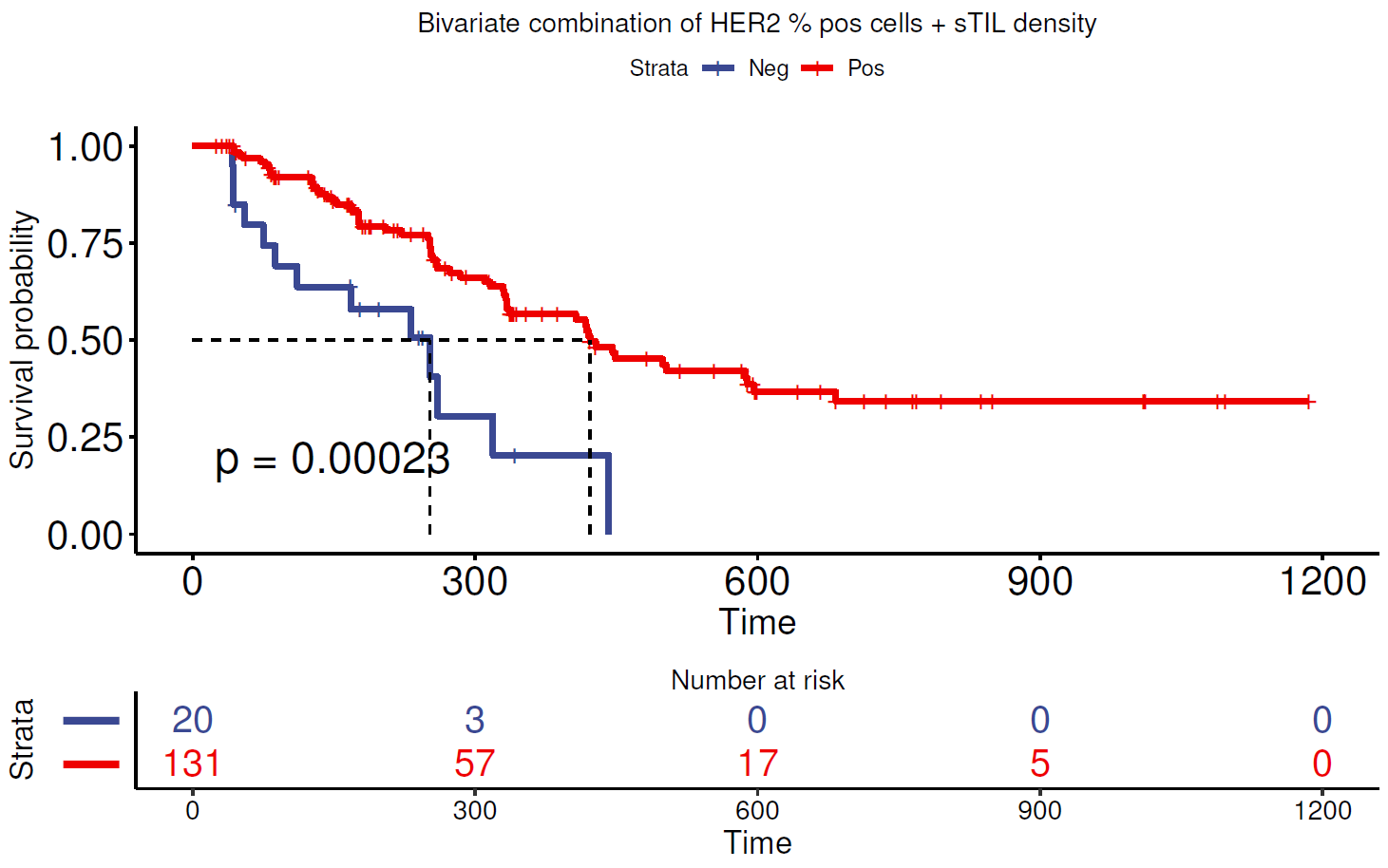


**B**
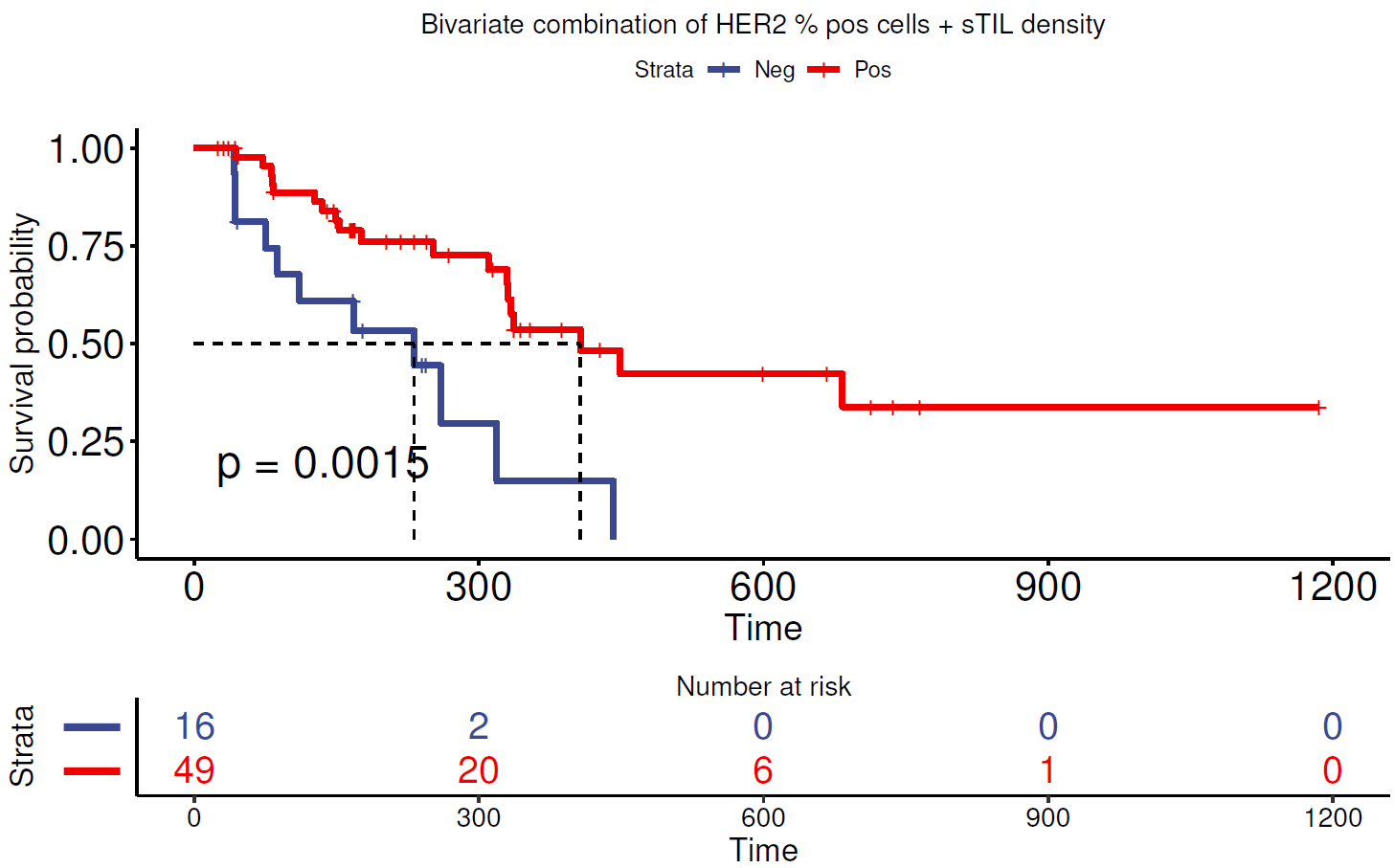


**C**
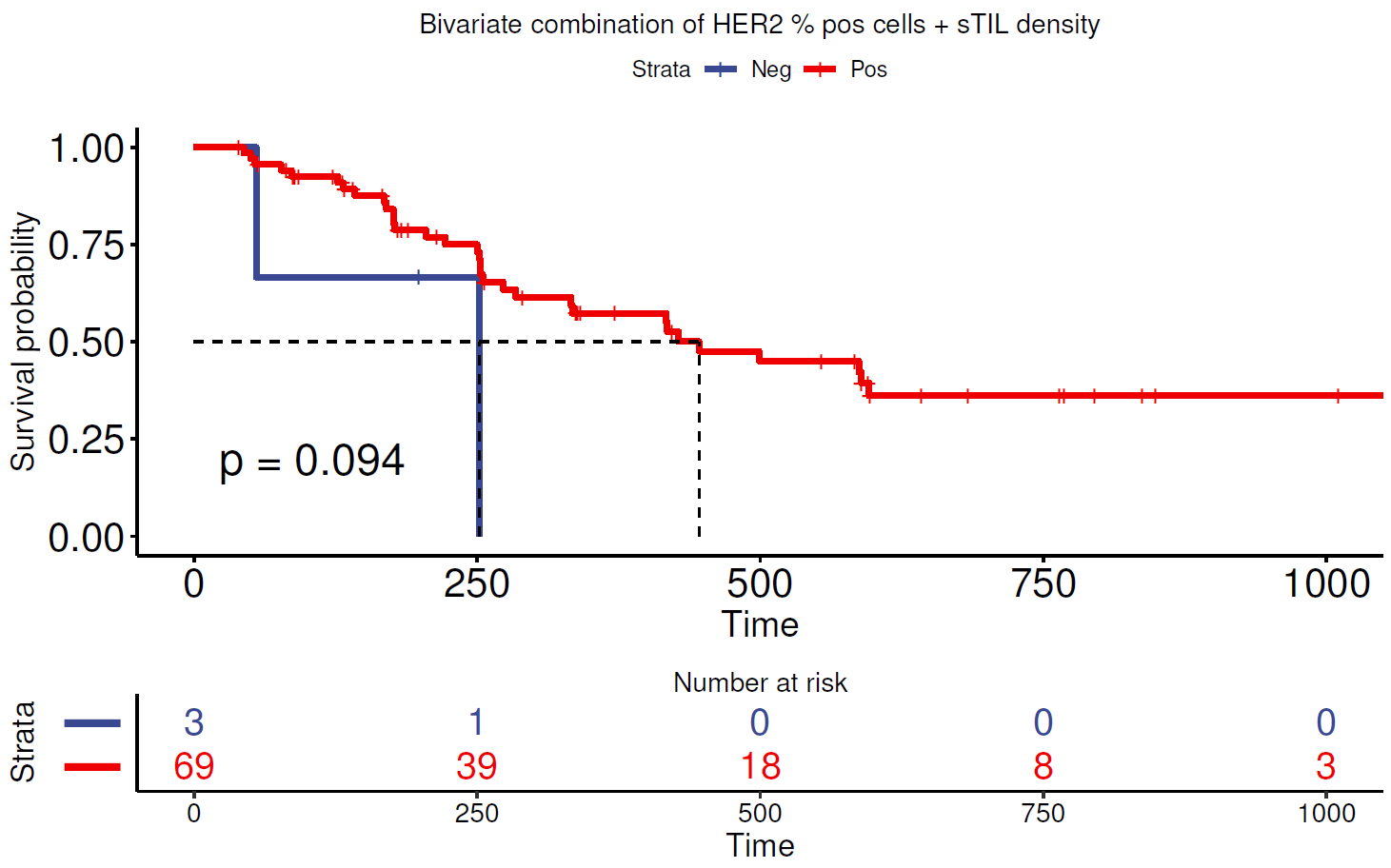


**D**
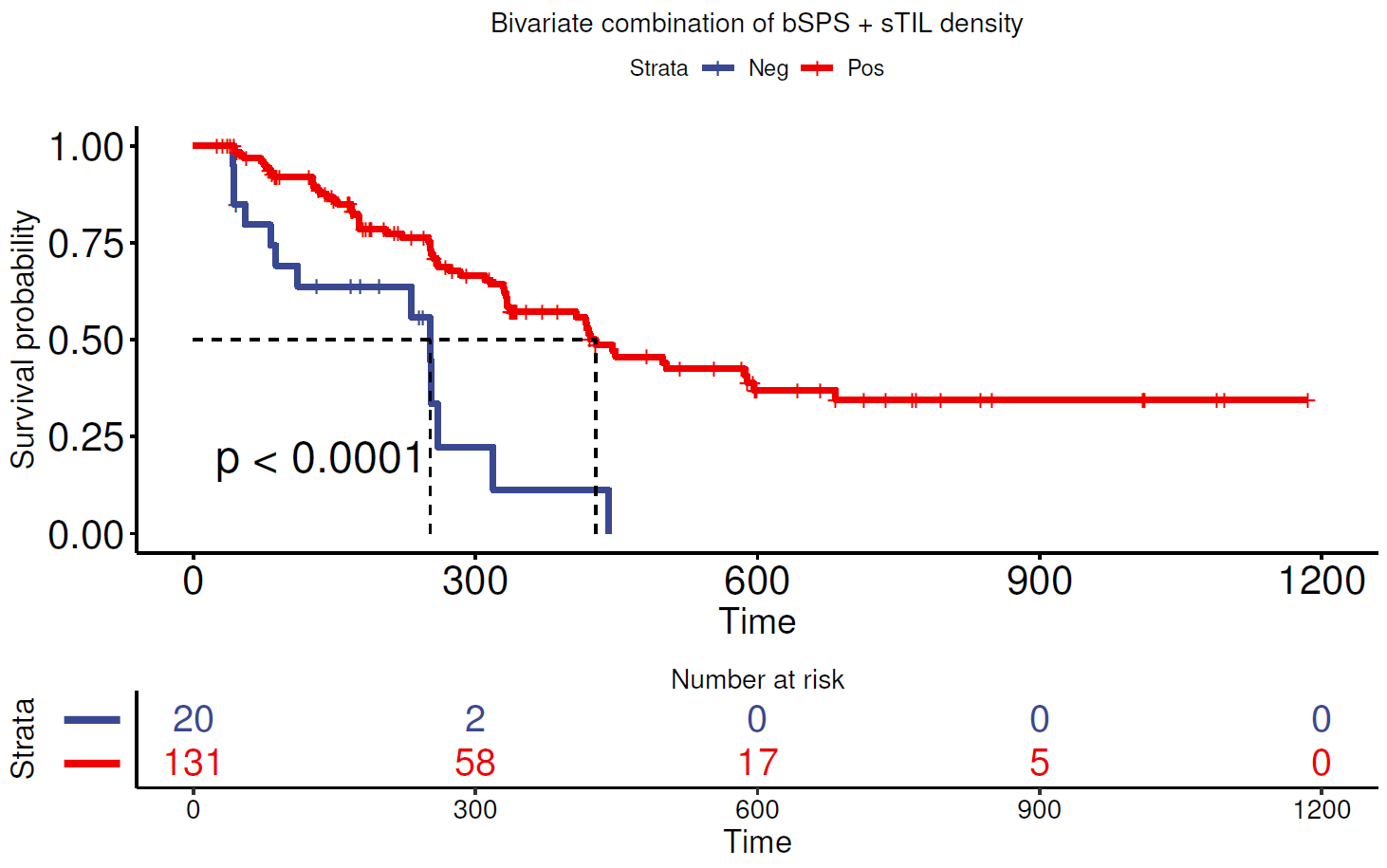


**E**
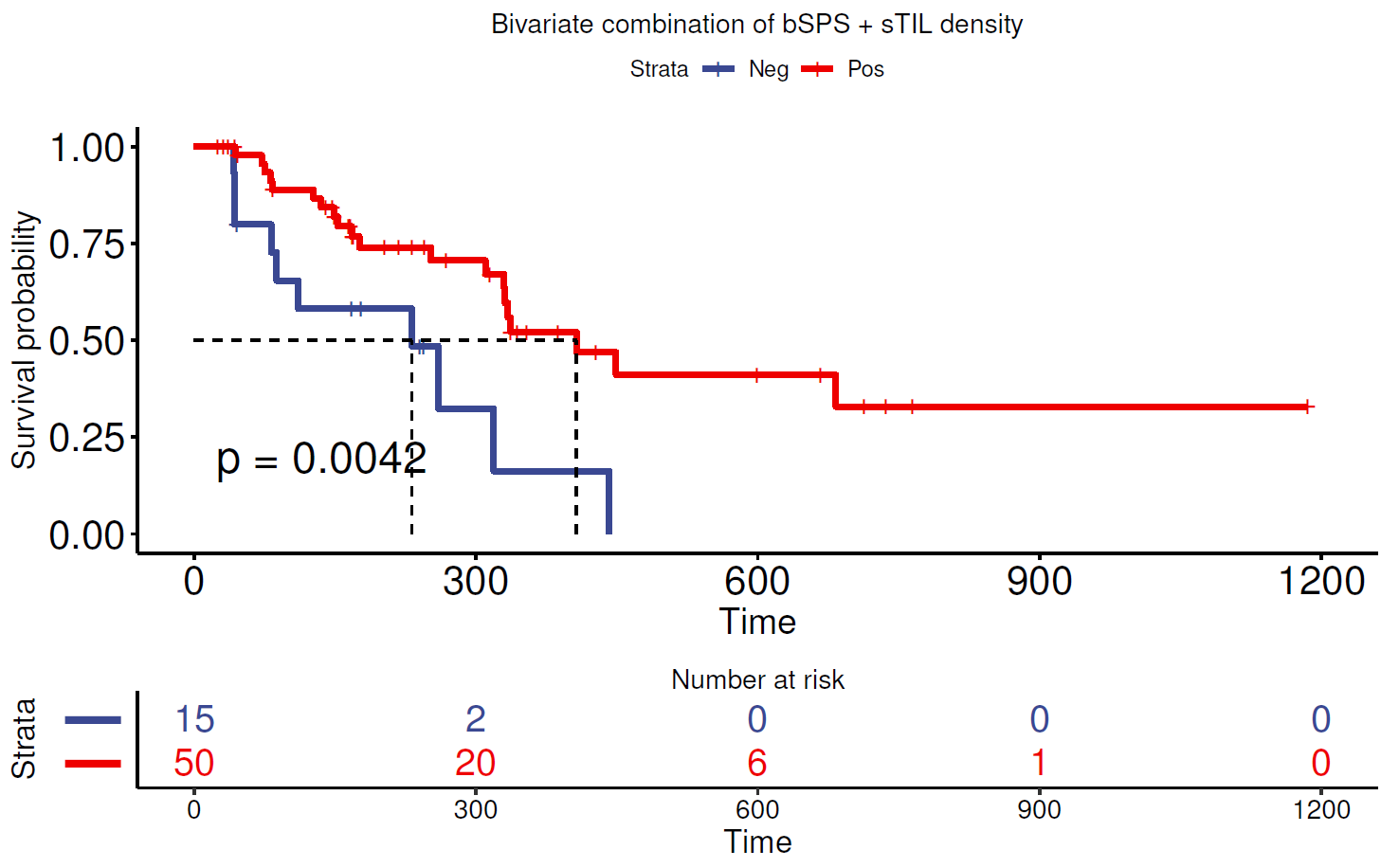


**F**
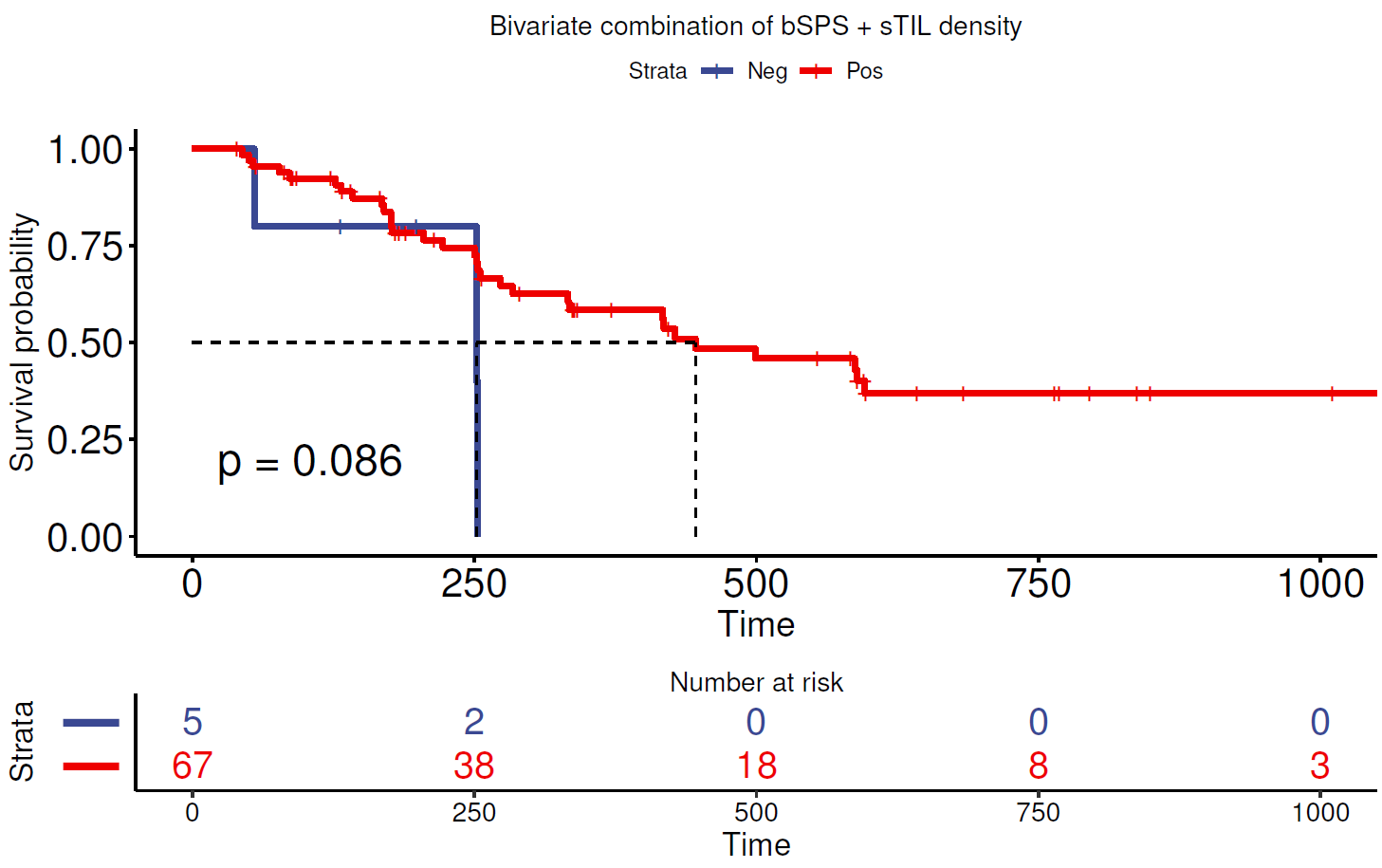


# Application of quantitative continuous scoring-based analysis on immuno-oncology–treated (anti–PD-L1–treated) clinical trial data

The pathologist’s visual assessment of tumor proportion score (TPS) on anti–programmed death-ligand 1 (PD-L1)–stained tissue samples is an established method to select patients with metastatic non-small cell lung cancer (NSCLC) who are likely to respond to anti–PD-L1 / PD-L1 monotherapy^3^. The quantitative continuous scoring (QCS)-based analysis of PD-L1 can redefine the PD-L1 TPS estimation by allowing PD-L1 to be scored in a more objective and quantifiable manner.

By applying the QCS-based image analysis pipeline using^4^ the epithelial segmentation model, the PD-L1 expression of each tumor cell compartment was estimated by the respective optical density (OD) of 3,3-diaminobenzidine (DAB), and tumor cells with a membrane OD greater than OD_threshold_ (refers to the OD threshold set to label a cell OD positive) were considered OD positive. A slide comprising a greater percentage of OD-positive tumor cells than a cut-point value was considered QCS positive (see Supplementary Fig. **S3**).

**Supplementary Figure S3.** PD-L1 QCS workflow that takes as an input a whole-slide image acquired from a PD-L1 IHC tissue section (left column), segments the tumor cells to membrane, cytoplasm, and nucleus (middle column), and derives OD-based readouts per cell that are aggregated as histograms (right column). Following cut-point optimization, a PD-L1 QCS-positive case is defined as one in which ≥57% of cells exhibit OD ≥8. IHC, immunohistochemistry; OD, optical density; PD-L1, programmed death-ligand 1; QCS, quantitative continuous scoring.


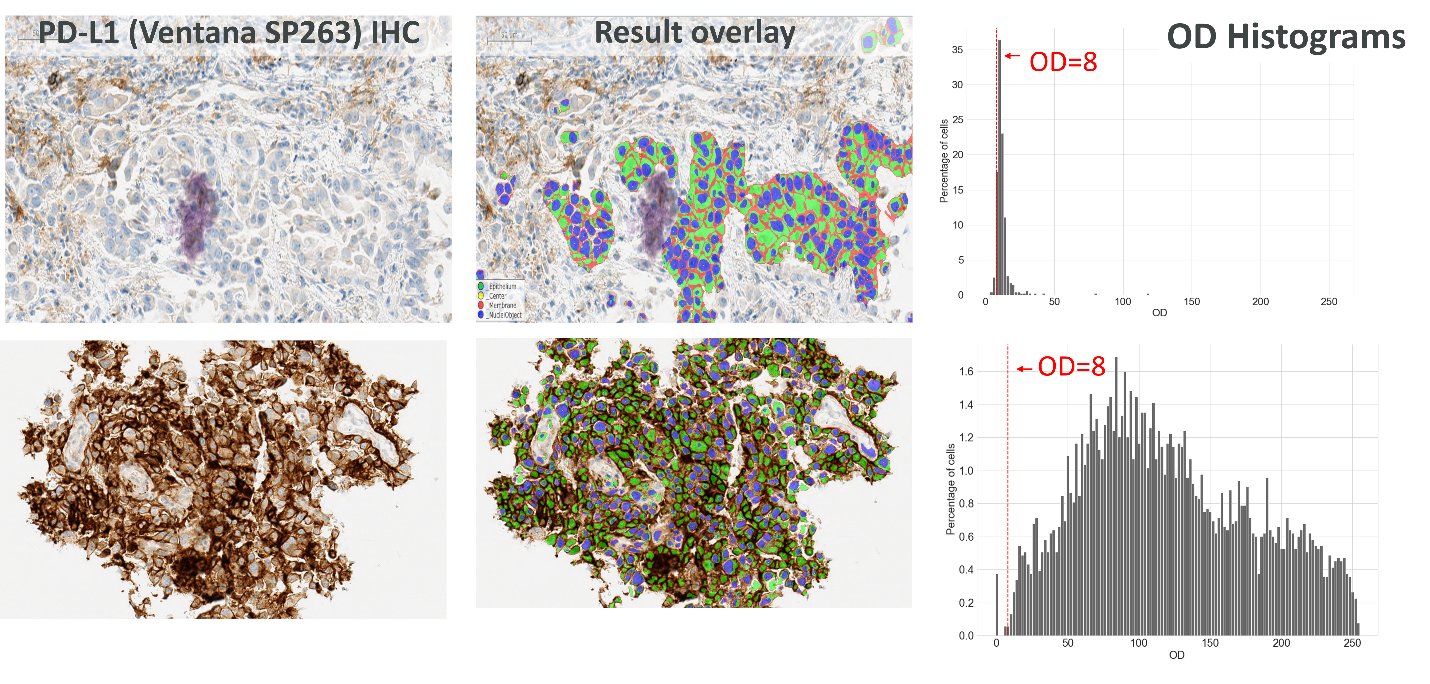


The OD_threshold_ and cut-point parameters were linked to patient overall survival by minimizing the Kaplan-Meier log–rank *p* values and keeping ≥50% prevalence in the QCS-positive subgroup. A PD-L1 QCS–based analysis (OD_threshold_=8, cut point ≥57%) stratifies durvalumab-treated patients with NSCLC at a higher prevalence and with a significant Kaplan-Meier log–rank *p* value (64%; *p*=0.0001) for overall survival compared with pathologist TPS (59%; *p*=0.01) (Supplementary Fig. **S**[**4**](#_bookmark38)). Median overall survival of 19.2 vs 7.9 months was observed in the QCS-positive vs -negative subgroup, respectively. The box plots (Supplementary Fig. **S5A**) indicate an overall good agreement (72% concordance) of the fully automated QCS with the pathologist TPS, which quantitatively supports the positive visual assessment of the cell segmentation accuracy. The grouped Kaplan-Meier (Supplementary Fig. **S5B** shows four arms based on stratification where QCS and pathologist TPS agree/disagree on the category. The survival of discordant cases favors the QCS for patient selection compared with pathologist TPS.

The novel QCS-based approach provides an objective way of correlating a quantitative estimate of PD-L1 immunohistochemistry (IHC) expression on tumor cells with survival of patients with NSCLC treated with durvalumab in later lines of therapy. An efficacy data-driven definition of PD-L1 tumor cell positivity may identify more patients benefiting from therapy with higher precision. Further validation of the QCS-based approach is needed to correlate with outcomes in the first-line setting and in comparison with other PD-L1 cutoff levels.

**Supplementary Figure S4.** Kaplan-Meier curves for overall survival stratification with (left) manual tumor proportion score (25% cut point) and (right) automated quantitative continuous scoring (57% cut point).


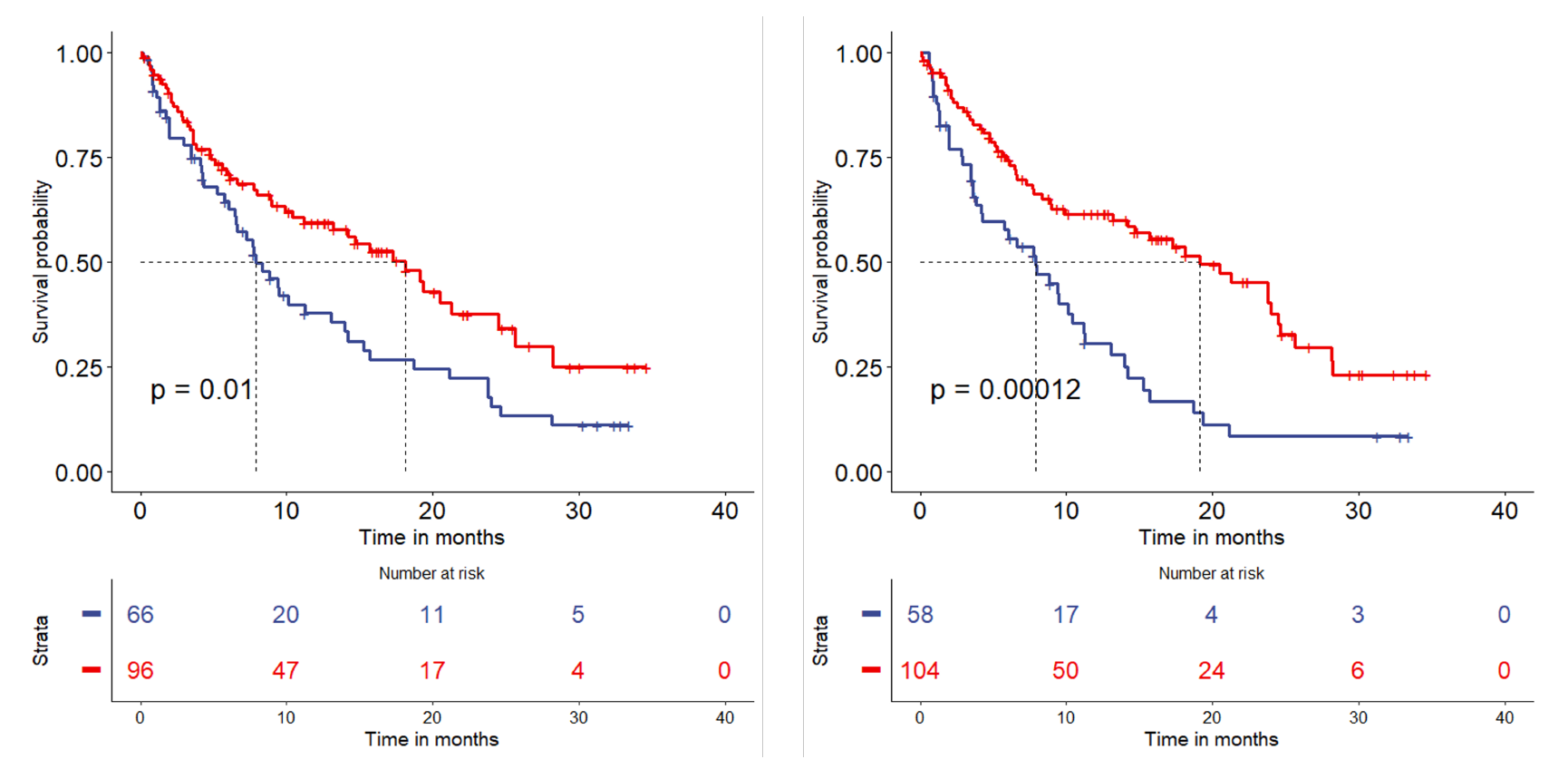


**Supplementary Figure S5.** Subgroup analysis. (**A**) Box plot indicating % OD-positive cells (OD ≥8) within the PD-L1–high and –low groups per pathologist assessment by TPS. (**B**) Grouped Kaplan-Meier curves showing stratification where QCS and pathologists agree/disagree on the scoring category. OD, optical density; Path, pathologist; PD-L1, programmed death-ligand 1; QCS, quantitative continuous scoring; TPS, tumor proportion score.

**A**
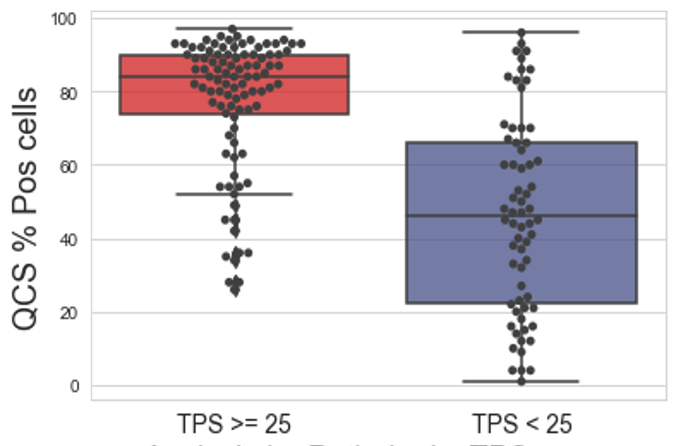


**B**

**
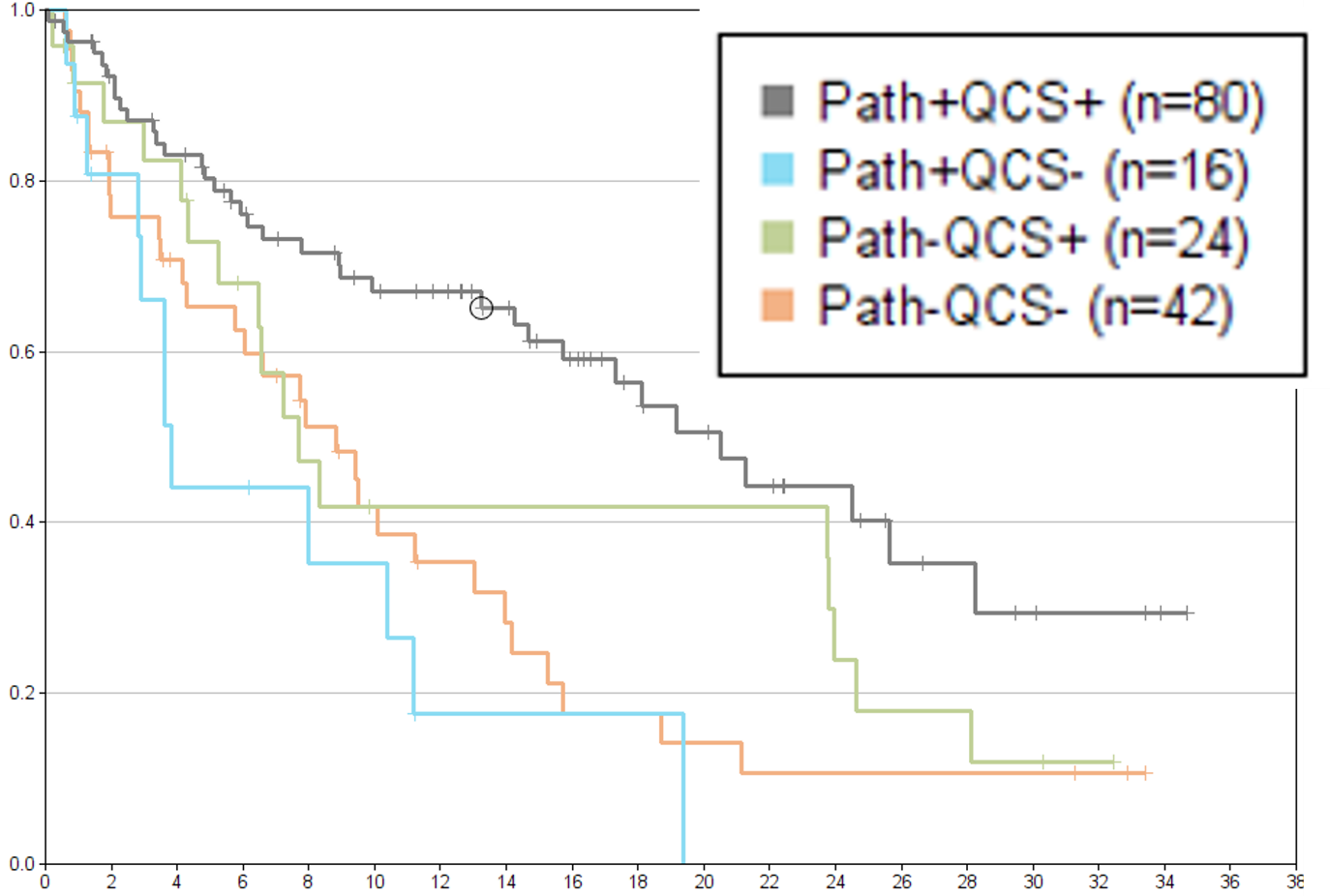
**

# Optical density computation

The human epidermal growth factor receptor 2 QCS quantifies DAB staining intensity generated by a human epidermal growth factor receptor 2 immunohistochemistry assay in individual cell compartments using the hue saturation density model^5^. Images are acquired using a red-green-blue color model that comprises 8-bit (256) gray values for each color channel. Using Beer-Lambert’s law, each color channel is converted to density using a logarithm and parameters *I*_0_, which are related to physical properties such as sectioning thickness and slide scanner illumination brightness. Based on those log-transformed channels, a color separation using the hue saturation density model estimates the density of the brown color from the DAB dye, which is referred to in the manuscript as the OD. An initial output in the range (0 *...* 5*.*75)^5^ is converted to the standard red-green-blue grayscale range (0 *...* 255) by applying a scaling factor.

**Supplementary Figure S6.** The hue saturation density model[^5^](#_bookmark31) estimates 3,3-diaminobenzidine (DAB) staining and, therefore, human epidermal growth factor receptor 2 (HER2) expression in each tumor cell as optical density. HRP, horseradish peroxidase; IHC, immunohistochemistry; RGB, red-green-blue.


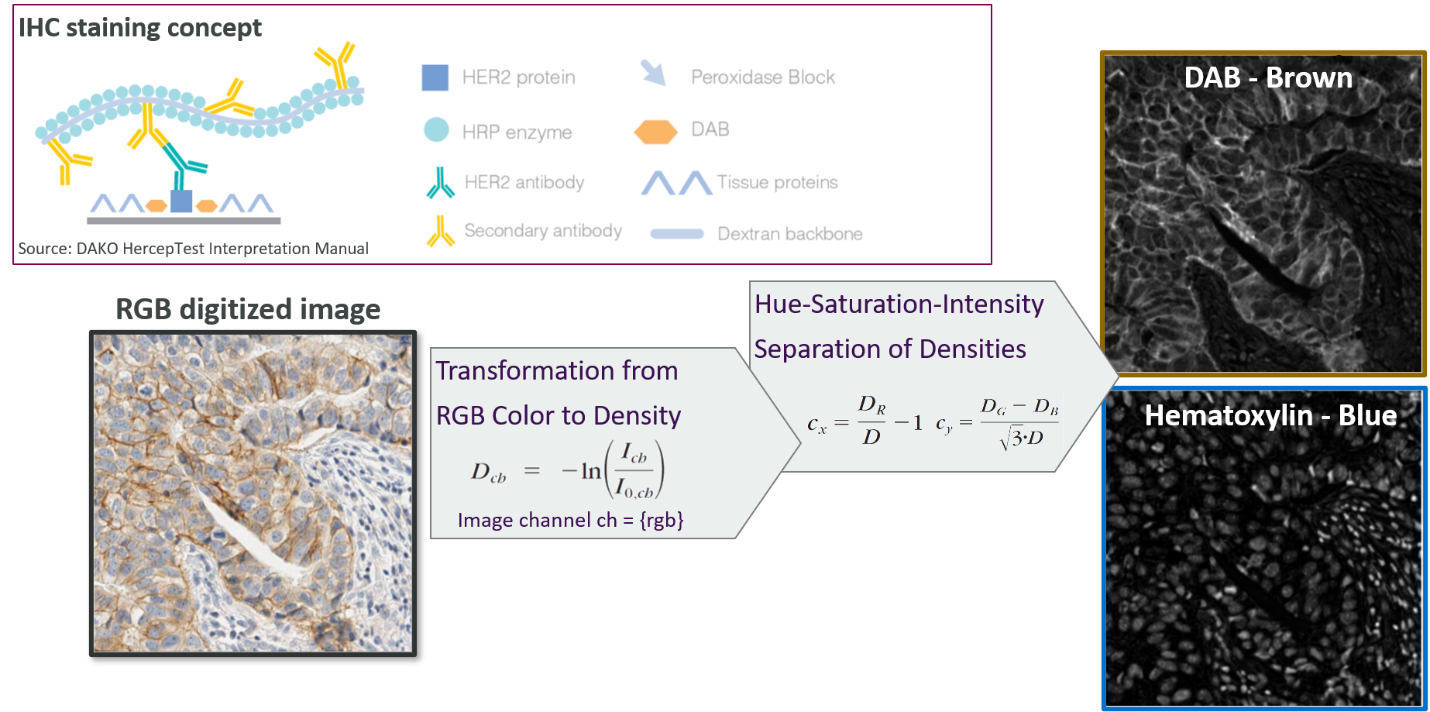


# Overlap between QCS binary Spatial Proximity Score and QCS %OD positive cells signatures.

The bSPS is a more generalized form of % OD-positive cells. The formulation of bSPS is as follows (Equation ([**3**](#_bookmark15)) in main text)

$$bSPS\left( r, OD_{threshold} \right)= \frac{\sum_{i} \mathbb{I[\exists}j\inͷ\left( r \right)_{i}:{OD}_{{membrane}^{j}}\geq OD_{threshold}}{N}$$

where N refers to the total number of detected tumor cells on the slide; Ni r refers to all the cells in the neighborhood of a cell *I* within a radius of *r*. The *OD_threshold_* refers to the OD threshold set to label a cell OD positive.

In this equation, with *r* → 0, the neighborhood reduces to the cell itself and becomes the formulation of %OD positive cells. Hence with the same *OD_threshold_*, the bSPS score will always be greater than or equal to %OD positive cells.

From the current study, the best signatures (feature + model parameters + cut-point) is found to be the following: a) bSPS score (*OD_threshold_* = 8, r=50$\mu m$) > 99.8 and b) %OD+ cells (*OD_threshold_* = 6) > 98.51. Please note that the *OD_threshold_* for the optimal signatures are different for the study. Nevertheless, high overlap in biomarker negative and positive population is still seen as seen in the confusion matrix below. It is seen that all %OD positive cases are contained within the BM+ cases determined by bSPS. There are 3 cases that are BM+ by bSPS but BM- by %OD positive cells, in which 2 out 3 patients are responders.

|  | %OD cells BM+  (OD=6) | %OD cells BM-  (OD=6) |
| --- | --- | --- |
| bSPS BM+  (r=50$\mu m$, OD=8) | 114 | 3 |
| bSPS BM-  (r=50$\mu m$, OD=8) | 0 | 34 |

# Ridge Plots for QCS cut-point stability analysis

To gauge the robustness of the best-performing features, we evaluated stratification performance as a function of both the cut point and the feature parameters using ridge plots (for the % OD-positive cells, see Fig. **S7A**) and bSPS (Fig. **S7B** and Fig. **S7C**). For the % OD-positive cells, different OD thresholds for cellular positivity were plotted. The bSPS has two parameters: the OD threshold and the radius. To this end, we created two ridge plots for bSPS: one keeping the OD threshold constant and plotting different radii and one keeping the radius constant and plotting different OD thresholds. Additional QCS-based features exploring the relationship between membrane and cytoplasm were also explored but did not relate to a significant correlation with ORR or PFS.

**Supplementary Figure S7.** Ridge plots indicating cut point stability for top-performing human epidermal growth factor receptor 2 (HER2) quantitative continuous scoring (QCS)-based features. Each point on each line in the ridge plot corresponds to a cut point for patient stratification for a particular parameter set of the QCS feature. The “deeper” the point within the “valley”, the lower the progression-free survival (PFS) log-rank *p* value. The accepted prevalence range (20–80%) is indicated by the white background areas on each plot. The broad and deep valleys in the ridge plots for both % optical density (OD)-positive cells and binary spatial proximity scores (bSPS) indicate robust cut points (i.e., a nearby cut point could be selected for stratification while maintaining significant *p* values). This suggests an increased likelihood for these features to preserve significance even on new cohorts. (**A**) Ridge plot showing stability of percentage of OD-positive cells at different OD thresholds. (**B**) Ridge plot showing stability of bSPS at R=50 µm and at different OD thresholds. (**C**) Ridge plot showing stability of bSPS across different radii using membrane OD ≥8.

**A

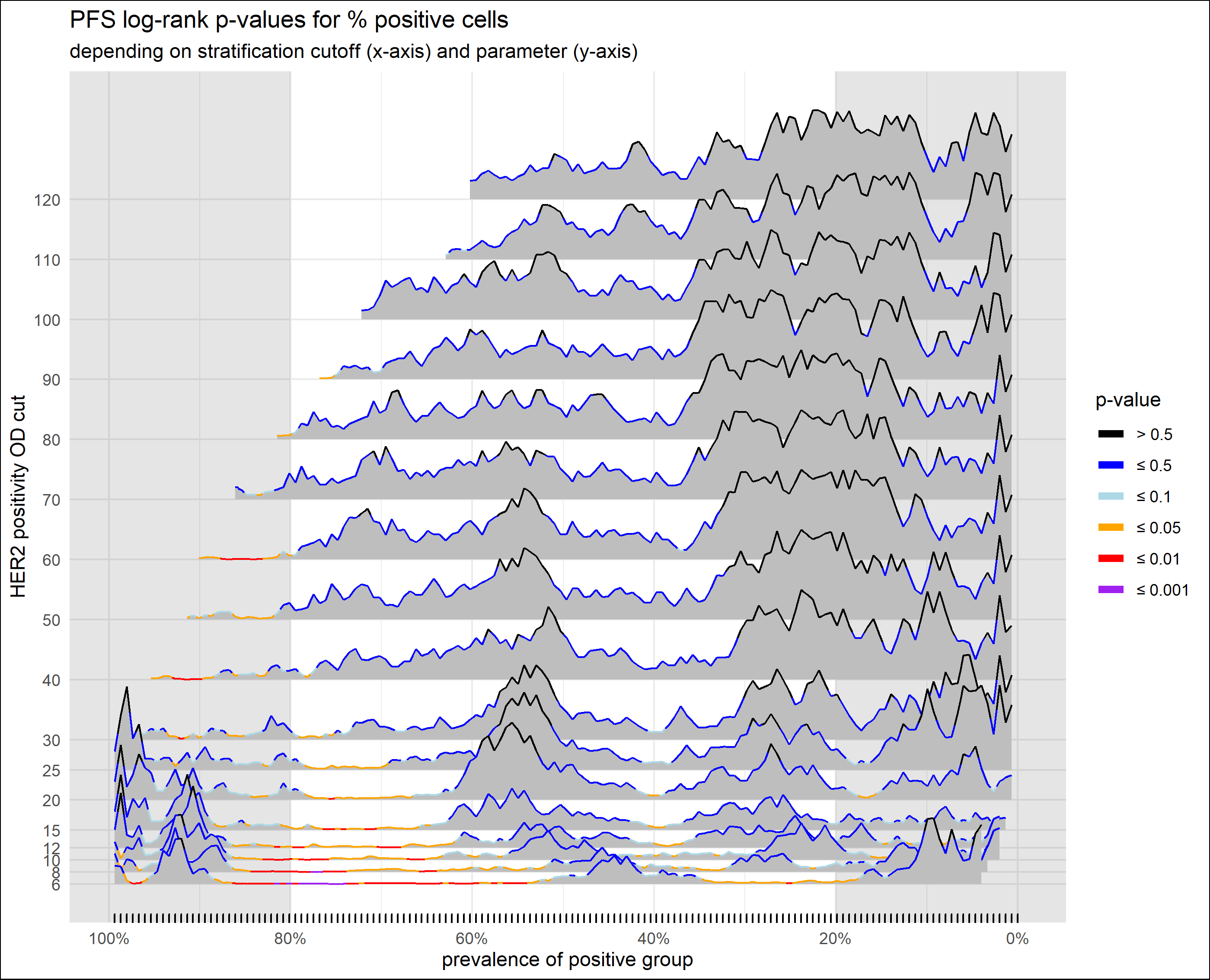
**

**B

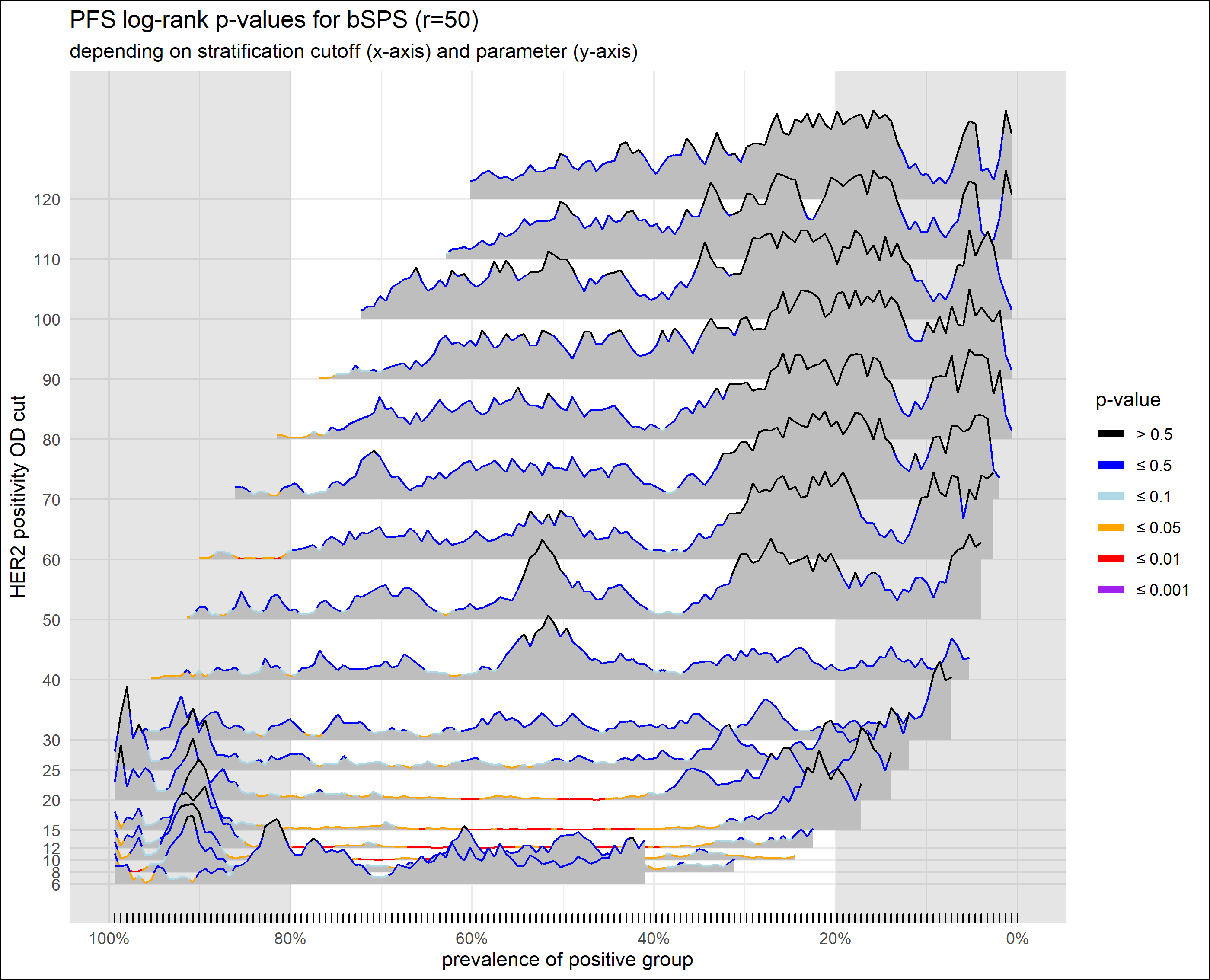
**

**C

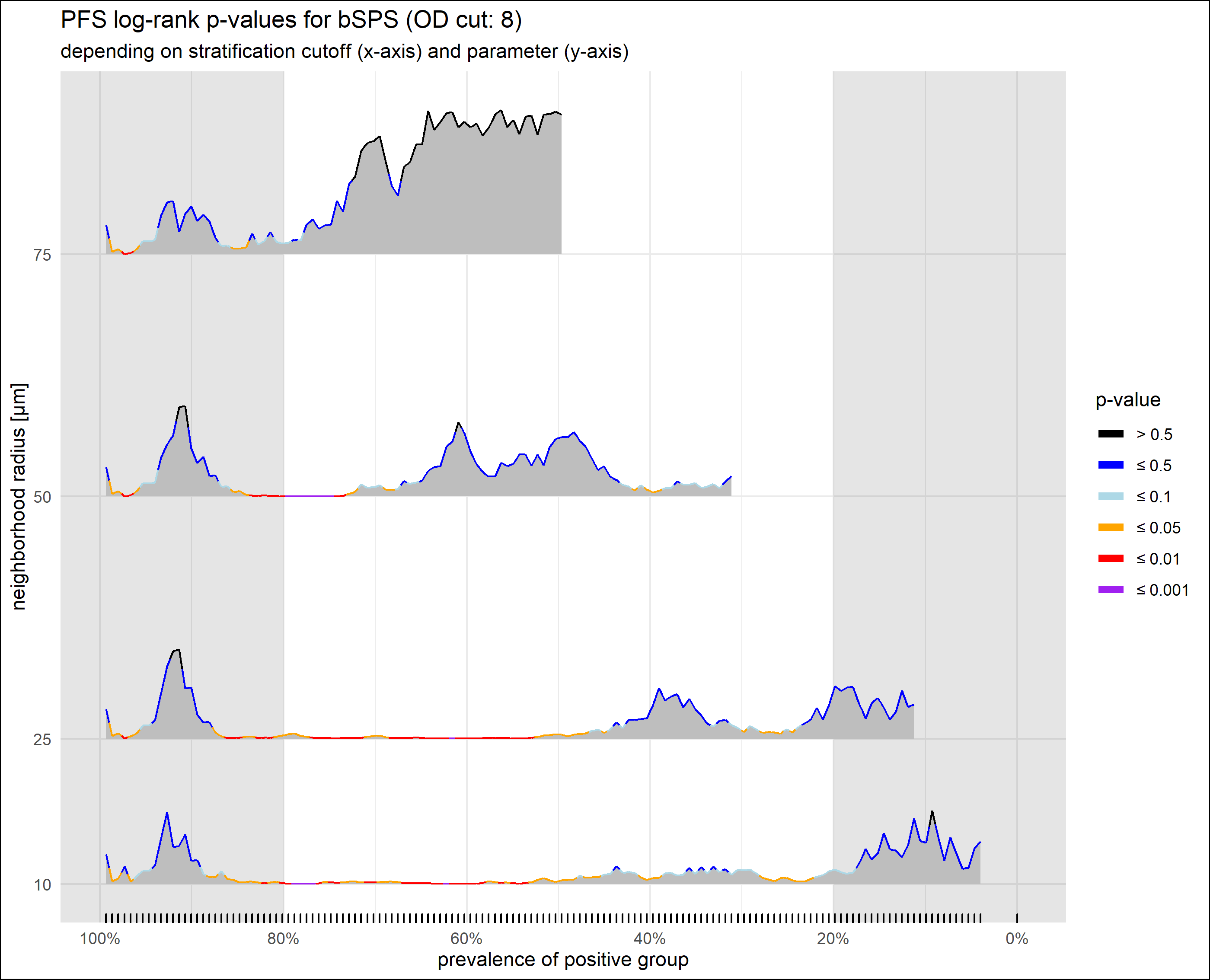
**

## Combination features of HER2 QCS and TIL features

The striking differences between TIL-based and HER2 QCS-based features when comparing HER2-positive and HER2-low subgroups led to the question of whether a combination of both feature types could further improve predictions. Generally, multivariate combinations of two or more features can quickly lead to a combinatorial explosion of hypotheses to be tested. This could potentially lead to a substantial lack in statistical power. Therefore, we decided to focus only on the top-two ranked HER2 QCS-based features and evaluated their correlation to the sTIL density. Neither the % OD-positive cells nor the bSPS were correlated with TIL densities (Spearman correlations approximately −0.1 for both), thus adding complementary information to the respective combination models. Stratification results with feature combinations are summarized in Table **S4** (see also Supplementary Fig. S2). We see that for both combinations, the prevalence increased from their univariate counterparts, and at the same time, the log-rank *p* values decreased.

# References

1. Wolff, A. C. *et al.* Human epidermal growth factor receptor 2 testing in breast cancer: American Society of Clinical Oncology/College of American Pathologists clinical practice guideline focused update. *Arch. Pathol. Lab. Med.* **142**, 1364–1382 (2018).

2. Miglietta, F. *et al.* Evolution of HER2-low expression from primary to recurrent breast cancer. *NPJ Breast Cancer* **7**, 137 (2021).

3. Rebelatto, M. C. *et al*. Development of a programmed cell death ligand-1 immunohistochemical assay validated for analysis of non-small cell lung cancer and head and neck squamous cell carcinoma. *Diagn. Pathol.* **11**, 95 (2016).

4. Kapil, A. *et al*. Domain adaptation-based deep learning for automated tumor cell (TC) scoring and survival analysis on PD-L1 stained tissue images. *IEEE Trans. Med. Imaging* **40**, 2513–2523 (2021).

5. Van der Laak, J. A., Pahlplatz, M. M., Hanselaar, A. G. & de Wilde, P. C.
Hue-saturation-density (HSD) model for stain recognition in digital images from transmitted light microscopy. *Cytometry* **39**, 275–284 (2000).

6. Yeghiazaryan V, Voiculescu I. Family of boundary overlap metrics for the evaluation of medical image segmentation. J Med Imaging (Bellingham). 2018 Jan;5(1):015006. doi: 10.1117/1.JMI.5.1.015006. Epub 2018 Feb 19. PMID: 29487883; PMCID: PMC5817231.
